# Bacterial Template Synthesis of Multifunctional Nanospindles for Glutathione Detection and Enhanced Cancer-Specific Chemo-Chemodynamic Therapy

**DOI:** 10.34133/2020/9301215

**Published:** 2020-03-26

**Authors:** Yan-Wen Bao, Xian-Wu Hua, Jia Zeng, Fu-Gen Wu

**Affiliations:** State Key Laboratory of Bioelectronics, School of Biological Science and Medical Engineering, Southeast University, 2 Sipailou Road, Nanjing 210096, China

## Abstract

Biological synthetic methods of nanoparticles have shown great advantages, such as environmental friendliness, low cost, mild reaction conditions, and enhanced biocompatibility and stability of products. Bacteria, as one of the most important living organisms, have been utilized as bioreducing nanofactories to biosynthesize many metal nanoparticles or compounds. Here, inspired by the disinfection process of KMnO_4_, we for the first time introduce bacteria as both the template and the reducing agent to construct a novel tumor microenvironment-responsive MnO_*x*_-based nanoplatform for biomedical applications in various aspects. It is found that the bacterium/MnO_*x*_-based nanospindles (EM NSs) can efficiently encapsulate the chemotherapeutic agent doxorubicin (DOX), leading to the fluorescence quenching of the drug. The as-formed DOX-loaded EM NSs (EMD NSs) are proven to be decomposed by glutathione (GSH) and can simultaneously release DOX and Mn^2+^ ions. The former can be utilized for sensitive fluorescence-based GSH sensing with a limit of detection as low as 0.28 *μ*M and selective cancer therapy, while the latter plays important roles in GSH-activated magnetic resonance imaging and chemodynamic therapy. We also demonstrate that these nanospindles can generate oxygen in the presence of endogenous hydrogen peroxide to inhibit P-glycoprotein expression under hypoxia and can achieve excellent tumor eradication and tumor metastasis inhibition performance. Taken together, this work designs a multifunctional bacterially synthesized nanomissile for imaging-guided tumor-specific chemo-chemodynamic combination therapy and will have implications for the design of microorganism-derived smart nanomedicines.

## 1. Introduction

Chemotherapy is one of the most common strategies employed in oncology [1]. Doxorubicin (DOX), a United States Food and Drug Administration-approved anthracycline antibiotic, is clinically used as a chemotherapeutic agent for the treatment of various malignant tumors [2, 3]. However, its short systemic circulation time, undesirable biodistribution, and nonspecific toxicities to cancer/normal cells lead to not only relatively low anticancer efficacy but also severe side effects [4, 5]. To address these issues, various nanocarriers such as liposomes [6], micelles [7], dendrimers [8], metal–organic frameworks (MOFs) [9], carbon nanomaterials [10], and gold nanomaterials [11, 12] have been designed for the efficient delivery of DOX to cancer cells. Among them, conventional nanocarriers which tend to release the encapsulated DOX molecules passively cannot realize controllable drug release and fail to achieve satisfactory therapeutic efficacy [13]. Therefore, increasing efforts have been devoted to designing intelligent nanocarriers for drug delivery, which can respond sensitively to internal/external stimuli (e.g., pH, redox, enzymes, temperature, ultrasound, and light) for on-demand imaging/therapy [14–18].

It is well known that tumor microenvironment (TME) has unique physiological characteristics such as acidic pH, hypoxia, and elevated levels of hydrogen peroxide (H_2_O_2_) and glutathione (GSH) [18, 19]. Hypoxia, resulting from an imbalance between oxygen (O_2_) supply and consumption, can directly lead to the resistance of cancer cells to chemotherapy, photodynamic therapy, or radiotherapy [20–24]. Hypoxic adaptation is mainly mediated by a family of transcriptional regulators called hypoxia-inducible factors (HIFs), including HIF-1*α*, which is involved in angiogenesis, invasion, and metastasis of cancer cells [25–27]. The upregulated HIF-1*α* has been reported to be associated with the overexpression of P-glycoprotein (P-gp), an important protein of the plasma membrane that recognizes external therapeutic agents and pumps them out of the cells, resulting in poor therapeutic outcomes [21, 28, 29]. The GSH level in cancer cells/tissues is reported to be higher than that in normal cells/tissues [16, 30–32], and hence, GSH may represent an important signal molecule to improve the specificity of cancer diagnosis and therapy. Besides, the overexpression of GSH in cancer cells can consume the reactive oxygen species (ROS), thus protecting the cancer cells [33–35]. Therefore, it is highly desirable to develop a TME-responsive nanosystem with selective drug release, GSH depletion, and O_2_ generation capabilities for efficient cancer therapy.

In nanotechnology, there are mainly three approaches for nanoparticle fabrication including physical, chemical, and biological methods. Compared with the former two traditional approaches, biosynthesis, during which nanoparticles are synthesized within the living or dead organisms, is mild, environmentally friendly, and usually inexpensive [36]. Importantly, the biosynthesized nanoparticles exhibit enhanced biocompatibility and stability. Various biological organisms have been utilized to biosynthesize many metal nanoparticles or compounds [37, 38]. For instance, Cui et al. synthesized the fluorescent CdSe quantum dots using living yeast cells as a controllable biosynthesizer [39]. Tian et al. biofabricated the selenium-containing nanoparticles in *Shewanella oneidensis* MR-1 cells using an extracellular electron transfer regulation strategy [40]. Bacteria, as one of the most important living organisms, are considered as the optimal bioreducing nanofactories due to their simple culturing conditions and rapid propagation [41]. However, there has been almost no research on the bacterium-biosynthesized manganese- (Mn-) containing nanoparticles.

In recent years, MnO_*x*_-based nanostructures have attracted substantial attention as a unique type of TME-responsive theranostic agents [42–46]. Among them, manganese dioxide (MnO_2_) nanosheets have been reported to efficiently quench the fluorescence (FL) emission of adsorbed fluorophores, and the quenching effect will be reversed by GSH, which can be utilized for the quantification of GSH concentration [47–50]. On the other hand, it has been reported that MnO_*x*_-based nanostructures can be decomposed by reaction with either H^+^ or GSH in the TME, generating Mn^2+^ ions that can significantly enhance *T*_1_-magnetic resonance (MR) imaging contrast for tumor-specific imaging and detection [48, 51]. Meanwhile, MnO_2_ nanostructures are able to trigger the decomposition of H_2_O_2_ existing in the TME into water and O_2_, so as to relieve tumor hypoxia [52]. Additionally, chemodynamic therapy (CDT), as an emerging therapeutic strategy using the in situ Fenton reaction or Fenton-like reaction to generate hydroxyl radical (^·^OH) to induce cell apoptosis, has attracted much attention due to its high selectivity and lethality activated by internal stimuli [53–57]. Apart from the commonly used iron ions that can induce Fenton reaction, other metal ions including Mn^2+^, Co^2+^, and Cu^2+^ also show Fenton-like activity [58, 59], which can be developed to be new types of chemodynamic nanoagents.

**Figure 1:**
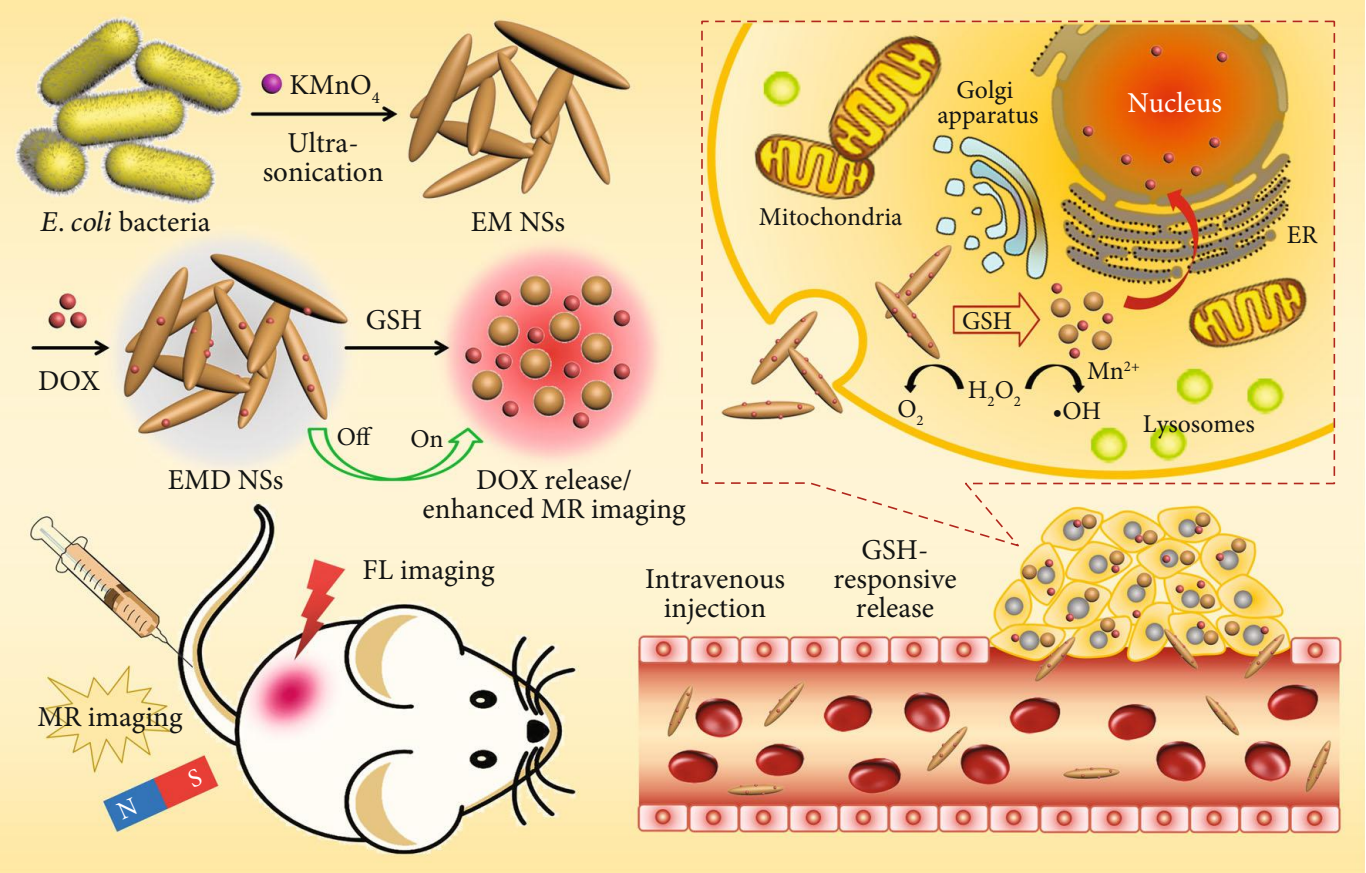
Schematic illustration of the fabrication of TME-responsive EMD NSs and their applications in rapid GSH detection and enhanced chemo-chemodynamic combination therapy for efficient tumor ablation.

In this work, inspired by the disinfection process of the strong oxidizer potassium permanganate (KMnO_4_), during which MnO_4_^−^ is rapidly reduced to MnO_2_ accompanied by the release of ROS that can kill bacteria [60], we put forward a novel biosynthetic strategy to construct a TME-responsive MnO_*x*_-based nanoplatform for cancer theranostics (Figure 1). Specifically, *Escherichia coli* (*E. coli*) bacterial cells are directly used as both the template and the reducing agent to react with the KMnO_4_ aqueous solution via ultrasonication at room temperature. The as-formed nanomaterials possess the spindle-like morphology and are accordingly named as *E. coli*-MnO_*x*_ nanospindles (abbreviated as EM NSs). After DOX loading, the resultant EM NSs–DOX (abbreviated as EMD NSs) show GSH-triggered DOX release for rapid GSH detection and GSH depletion-enhanced chemotherapy, as well as Fenton-like Mn^2+^ delivery for enhanced MR imaging and CDT. Meanwhile, EMD NSs can react with H_2_O_2_ to generate a significant amount of O_2_, overcoming hypoxia-induced chemoresistance. Last but not least, we also demonstrate that EMD NSs can not only effectively eliminate the primary tumors but also inhibit tumor metastasis. To the best of our knowledge, there have been few reports that realize simultaneously the TME-responsive chemotherapy and the Fenton-like metal-based CDT. Besides, the present work may also represent the first example of fabricating microorganism-derived and GSH-responsive smart nanomedicines.

## 2. Results and Discussion

### 2.1. Preparation and Characterization of EM NSs and EMD NSs

**Figure 2:**
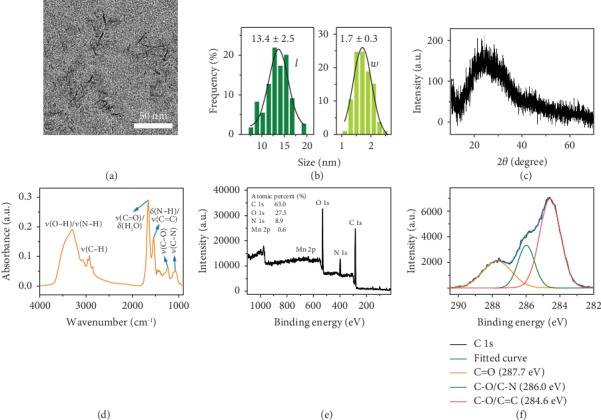
(a) TEM image of EM NSs. An enlarged version of this image was given in [Supplementary-material supplementary-material-1] for clearer observation. (b) Corresponding length (*l*) and width (*w*) histograms. (c) Powder XRD pattern of EM NSs. (d) FTIR spectrum of EM NSs. (e) XPS total and (f) high-resolution C 1s curves of EM NSs.

EM NSs were facilely prepared via the room-temperature ultrasonication of KMnO_4_-treated *E. coli* cells followed by purification. As it was observed that the suspension turned from purple to red in the KMnO_4_ incubation process, we considered that the structure of the bacterial cells was destroyed and the Mn element was completely or partially reduced. In the ultrasonication process, the Mn element might be further reduced and embedded in the oxidized bacterial fragments to form the final nanoparticles. As shown in the transmission electron microscopy (TEM) results (Figures 2(a) and 2(b), and [Supplementary-material supplementary-material-1]), the obtained EM NSs presented the spindle-like morphology with an average length of ~13.4 nm and an average width of ~1.7 nm, which were both smaller than the average hydrodynamic size (~31.0 nm, measured by dynamic light scattering (DLS); see [Supplementary-material supplementary-material-1]) due to the presence of hydration layers. The rod-shaped *E. coli* bacterial cells were chosen because they are Gram-negative bacteria with cell walls composed of thin peptidoglycan layers, which may be destructed after KMnO_4_ and ultrasonication treatments, resulting in the formation of the spindle-like nanoparticles. We also selected the rod-shaped *Bacillus subtilis* (*B*. *subtilis*) and spherical *Staphylococcus aureus* (*S*. *aureus*) cells, the two kinds of Gram-positive bacteria with thick cell walls, and tried to prepare nanomaterials via similar KMnO_4_ and ultrasonication treatments. Unfortunately, after these treatments, large bacterial cells were still observed in the obtained suspensions (Figures [Supplementary-material supplementary-material-1] and [Supplementary-material supplementary-material-1]), indicating that the short-time treatments could not completely destroy the Gram-positive bacteria. Additionally, when *B*. *subtilis* and *S*. *aureus* cells were preincubated with 1% Triton X-100 solution to increase the membrane permeability and then treated with the same KMnO_4_ and ultrasonication, the spindle- and dot-like nanostructures with sizes of ~13.5 × 2.2 nm and ~1.7 nm were obtained, respectively (Figures [Supplementary-material supplementary-material-1] and [Supplementary-material supplementary-material-1]). Because the rod-shaped bacteria (*E*. *coli* and *B*. *subtilis*) and the spherical bacteria (*S*. *aureus*) could be used to produce nanospindles and nanodots, respectively, we assume that the bacterial morphology affects the morphology of the synthesized nanostructures.

To date, several synthetic strategies have been proposed to prepare MnO_*x*_-based nanomaterials with different sizes and morphologies, including the thermal decomposition method [61], exfoliation strategy [62], adsorption-oxidation method [63, 64], hydro-/solvothermal method [65], and permanganate reduction method [66, 67]. These methods show their intrinsic advantages and disadvantages [68]. For example, the thermal decomposition strategy can precisely regulate the particle size and morphology but the oily solvent-capping surface limits the biomedical applications of the products. The exfoliation strategy can successfully obtain two-dimensional materials, but its synthetic process is harsh and time-consuming. The particular preparation conditions of adsorption-oxidation and hydro-/solvothermal methods may also restrict their wide applications. In this work, the preparation process of EM NSs can be classified as the permanganate reduction method, which is fast, simple, and widely adopted to produce the MnO_*x*_-based nanomaterials with good water dispersibility. Compared with the reported permanganate reduction methods, we creatively utilized the living bacterial cells as the reductant in this work, which has the following advantages: First, the bacterial cells as the raw materials are easily available and can be easily scaled up. Second, the whole bacterial cells are directly used as both the template and the reducing agent for the biosynthesis, without the extra steps such as the extraction of the cell walls/membranes. Third, this bacterial template synthesis is suitable for a variety of bacterial cells including Gram-negative and Gram-positive bacteria to generate the MnO_*x*_-based nanomaterials with unique morphologies. Finally, the dose of KMnO_4_ is low, which may ensure the good biocompatibility of the obtained nanoparticles.

Considering that the nanospindles prepared from *E*. *coli* have simpler synthetic procedures (which do not require the Triton X-100 treatment) compared with other bacterium-derived nanostructures, we chose the *E*. *coli*-derived nanospindles (namely, EM NSs) for further investigation. The X-ray diffraction (XRD) pattern of EM NSs demonstrated that the nanomaterials were in the amorphous state (Figure 2(c)). Figure 2(d) shows the Fourier transform infrared (FTIR) spectrum of EM NSs. The broad band at ~3300 cm^−1^ originates from the stretching vibrations of O–H and N–H. The peaks at 1654, 1241, and 1077 cm^−1^ represent the stretching vibrations of the C=O (accompanied by the contribution from the bending vibration of H_2_O), C–O, and C–N groups, respectively. Besides, the peak at 1541 cm^−1^ can be assigned to the bending vibration of N–H and/or the stretching vibration of C=C. Additionally, it was found that the characteristic FTIR peaks of EM NSs and freeze-dried *E. coli* ([Supplementary-material supplementary-material-1]) overlapped to a large extent, indicating that the main components of the organic materials in the EM NSs originated from the bacteria. Four elements can be observed in the X-ray photoelectron spectroscopy (XPS) total curve of EM NSs including C 1s (63.0%), O 1s (27.5%), N 1s (8.9%), and Mn 2p (0.6%) (Figure 2(e)). The valence states of key elements in EM NSs were determined according to the high-resolution curves (Figure 2(f) and [Supplementary-material supplementary-material-1]). The XPS high-resolution Mn 2p curve shows that Mn 2p_3/2_ and Mn 2p_1/2_ peaks center at 642.3 and 654.1 eV ([Supplementary-material supplementary-material-1]), respectively, indicating that the main form of the Mn element is MnO_2_ [69]. However, since the XPS signals were very weak due to the very low Mn content in the EM NSs, we could not rule out the presence of other manganese oxides such as MnO_3_ and MnO in the NSs. In addition, the Mn element content in EM NSs was measured to be 8.6 wt% by inductively coupled plasma optical emission spectrometry (ICP-OES). The long-term stability of EM NSs was also tested in phosphate-buffered saline (PBS) or cell culture medium (Dulbecco's modified Eagle's medium (DMEM)), and no obvious changes of hydrodynamic size were observed within two weeks as measured by DLS ([Supplementary-material supplementary-material-1]).

**Figure 3:**
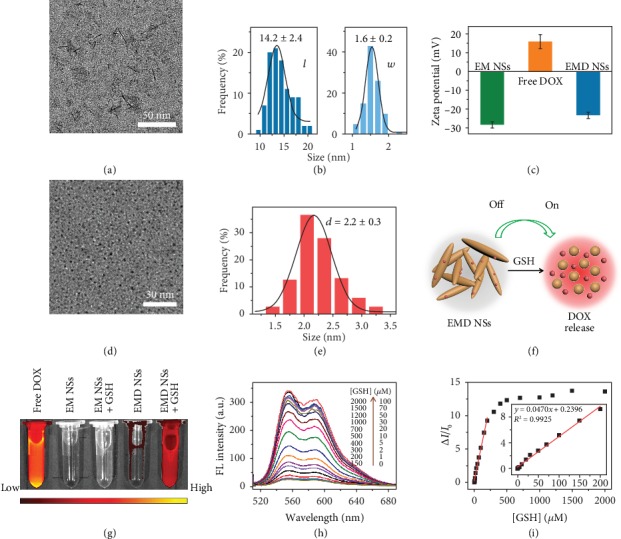
(a) TEM image of EMD NSs and (b) corresponding length (*l*) and width (*w*) histograms. An enlarged version of (a) was given in [Supplementary-material supplementary-material-1] for clearer observation. (c) Zeta potential results of EM NSs, free DOX, and EMD NSs. (d) TEM image and (e) corresponding size histogram of EMD NSs after incubation with GSH (2.0 mM). (f) Schematic illustration of EMD NSs before and after GSH incubation. (g) FL images of different solutions/suspensions as indicated. Ex = 500 nm and Em = 620 nm. (h) GSH concentration- ([GSH]-) dependent FL spectra of EMD NSs. Ex = 500 nm. (i) Relationship between Δ*I*/*I*_0_ (at 555 nm) and [GSH] (0–2000 *μ*M). Inset: linear fitting result of Δ*I*/*I*_0_ versus [GSH] in the [GSH] range of 0–200 *μ*M.

Then, DOX, which can simultaneously act as the therapeutic agent and the FL probe, was loaded into EM NSs via electrostatic and hydrophobic interactions to obtain the EMD NSs. The TEM image shows the unchanged spindle-like structure of EMD NSs with an average length of ~14.2 nm and an average width of ~1.6 nm (Figures 3(a) and 3(b) and [Supplementary-material supplementary-material-1]). The change of the zeta potential value from −28.5 ± 1.6 to −23.4 ± 1.5 mV (Figure 3(c)) and the characteristic absorption peak of DOX at ~490 nm in the ultraviolet–visible (UV–vis) spectrum of EMD NSs ([Supplementary-material supplementary-material-1]) further confirmed the successful loading of DOX molecules. The nanospindles exhibited an ultrahigh DOX encapsulation efficiency of ~100% and loading efficiency of 11.1%.

### 2.2. GSH Detection

Interestingly, the fluorescence of DOX was almost completely quenched in the formed EMD NSs via static quenching effect [50], and this MnO_*x*_-induced quenching effect can be reversed upon the addition of GSH ([Supplementary-material supplementary-material-1]). To investigate the effect of GSH on EMD NSs, TEM analysis of EMD NSs after GSH incubation was conducted. As shown in Figures 3(d) and 3(e), it can be clearly observed that GSH could degrade the MnO_*x*_-based nanospindles into smaller nanoparticles with an ultrasmall size of ~2.2 nm (which would promote deep tumor penetration and rapid renal clearance), accompanied by the release of DOX for cancer therapy. The GSH-responsive FL “turn on” process is schematically illustrated in Figure 3(f) and proven by the FL images (Figure 3(g)). Besides, the dissociation of EM NSs after GSH incubation demonstrated the destructive effect of GSH on the nanocarriers (EM NSs) ([Supplementary-material supplementary-material-1]), which could be due to the redox reaction of Mn element in high valence states in EM NSs, as shown in equation (1). We demonstrated that the “turn on” FL response of EMD NSs could be used for the sensitive and selective GSH detection. As shown in [Supplementary-material supplementary-material-1], after the addition of varied concentrations of GSH, the FL intensity of EMD NSs increased under the 365 nm UV irradiation. The GSH concentration-dependent FL enhancement of EMD NSs was also shown in the FL spectra under 500 nm excitation (Figure 3(h)). Figure 3(i) shows the corresponding change of FL intensity (Δ*I*/*I*_0_) at 555 nm as a function of GSH concentration ([GSH]). The relationship between Δ*I*/*I*_0_ and [GSH] could be described by the following equation when [GSH] is in the range of 0–200 *μ*M: Δ*I*/*I*_0_ = 0.2396 + 0.0470 × [GSH] (*R*^2^ = 0.9925), with a detection limit of 0.28 *μ*M (at a signal to noise ratio of 3). When [GSH] is higher than 200 *μ*M, the Δ*I*/*I*_0_ value increased nonlinearly with increasing [GSH]. Importantly, the response of EMD NSs was highly selective towards GSH relative to other substances (common ions and small molecules) in physiological solutions. As shown in [Supplementary-material supplementary-material-1], the FL intensity of EMD NSs markedly increased after GSH addition, while other substances such as ions (including K^+^, Na^+^, Ca^2+^, and Mg^2+^) and small molecules (including glucose, dithiothreitol (DTT), and amino acids like L-glutamic acid (Glu), glycine (Gly), L-serine (Ser), L-arginine (Arg), L-lysine (Lys), D,L-homocysteine (Hcy), and L-cysteine (Cys)) did not cause distinct FL enhancements, which was ascribed to the selective reducing ability of GSH towards the EMD NSs. It was worth noting that Hcy and Cys, the two biological thiols that may severely affect GSH detection, did not interfere with the GSH detection in our system.

(1) $$\begin{array}{c} {\text{MnO}}_{2}+2\text{GSH}+2{\text{H}}^{+}⟶{\text{Mn}}^{2+}+\text{GSSG}+2{\text{H}}_{2}\text{O} \end{array}$$

### 2.3. *In Vitro* O_2_ Generation

**Figure 4:**
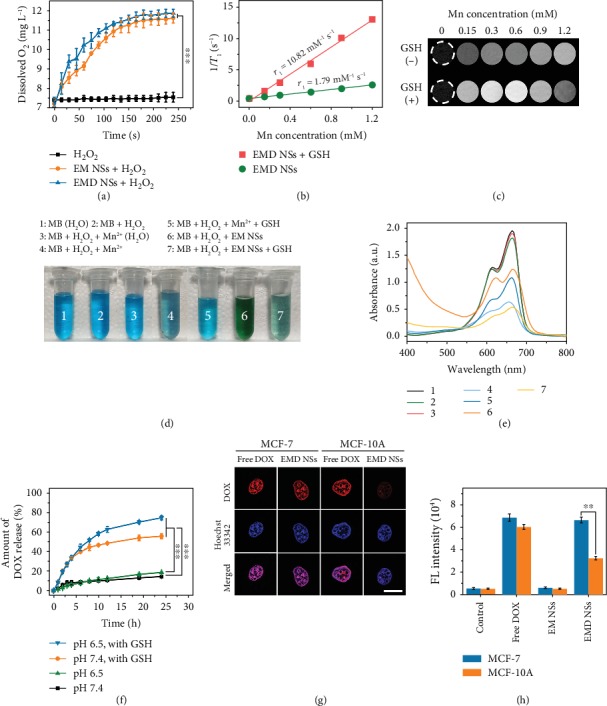
(a) O_2_ generation in H_2_O_2_ solutions (100 *μ*M) after addition of EM or EMD NSs (Mn concentration: 0.6 mM). (b) Linear fitting of 1/*T*_1_ versus Mn concentration for EMD NS suspensions and (c) *T*_1_-weighted MR images of EMD NS suspensions at varied Mn concentrations without (−) or with (+) GSH (2.0 mM). (d) Photographs and (e) UV–vis absorption spectra of MB-containing solutions after different treatments as indicated. The groups 1 and 3 were prepared in H_2_O, and the groups 2 and 4–7 were prepared in Na_2_CO_3_/NaHCO_3_ buffer solutions. (f) DOX release kinetics of EMD NSs in the presence or absence of GSH (2.0 mM) in solutions with different pH values (7.4 or 6.5). (g) Confocal images of MCF-7 cancer cells and MCF-10A normal cells after treatment with free DOX or EMD NSs followed by Hoechst 33342 staining (to visualize nuclei). (h) FL intensities of cells treated without (control) or with free DOX, EM NSs, and EMD NSs. ^∗∗^*P* < 0.01 and ^∗∗∗^*P* < 0.001.

Since MnO_2_ is known to be an excellent catalyst to trigger the decomposition of H_2_O_2_ into H_2_O and O_2_, we tested whether EM NSs and EMD NSs can generate O_2_ in vitro upon reaction with H_2_O_2_ at endogenous levels (e.g., 100 *μ*M). As shown in Figure 4(a), both EM NSs and EMD NSs could trigger rapid O_2_ generation within the H_2_O_2_ solution, demonstrating the catalytic property of EM NSs and EMD NSs to react with H_2_O_2_ and trigger its decomposition for O_2_ generation (equation (2)), relieving the tumor hypoxia.

(2) $$\begin{array}{c} {\text{MnO}}_{2}+{\text{H}}_{2}{\text{O}}_{2}+2{\text{H}}^{+}⟶{\text{Mn}}^{2+}+{\text{O}}_{2}+2{\text{H}}_{2}\text{O} \end{array}$$

### 2.4. MR Imaging, GSH Depletion, and Drug Release Behavior

Because the Mn element is an excellent *T*_1_-shortening agent in MR imaging, we investigated the ability of EMD NSs as GSH-activated MR imaging contrast agents. The in vitro longitudinal relaxation rate (1/*T*_1_) as a function of the Mn concentration of EMD NSs before and after GSH reduction was evaluated (Figures 4(b) and 4(c)). After GSH addition, the produced Mn^2+^ ions exhibited a stronger enhancement in *T*_1_-weighted MR imaging than the EMD NSs. Longitudinal relaxivity *r*_1_, obtained by measuring the relaxation rate as a function of Mn concentration, exhibited a 6-fold enhancement when EMD NSs were reduced to Mn^2+^ ions by GSH.

On the other hand, we selected methylene blue (MB), a dye that can be degraded by ^·^OH, as an indicator of ^·^OH generation. By comparing the results of groups 1–4 in Figures 4(d) and 4(e), we inferred that H_2_O_2_, Mn^2+^ ions, and carbonate/bicarbonate (CO_3_^2−^/HCO_3_^−^) buffer solution are all necessary to fade the blue MB solution, which was in accordance with the previous work [58]. Additionally, the results of groups 4 and 5 indicated that the added GSH eliminated the formed ^·^OH and inhibited the MB degradation. Based on the above results, we investigated the chemodynamic activity of our nanoagents. When Mn^2+^ ions were replaced by EM NSs (group 6), partial MB molecules degraded due to the catalytic activity of EM NSs. More importantly, in the presence of GSH, EM NSs exhibited an even higher MB degradation efficiency (group 7), which could be explained by the GSH-stimulated Mn^2+^ release from EM NSs accompanied with GSH depletion. These results indicated that the EMD NSs consisting of DOX and EM NSs can serve as both chemotherapeutic and chemodynamic nanoagents.

Furthermore, the in vitro drug release properties of EMD NSs in the presence or absence of GSH displayed the sustained and GSH-responsive DOX release from EMD NSs (Figure 4(f)), which is determined by the electrostatic interaction between positively charged DOX molecules and negatively charged EM NSs. Besides, at 24 h, the slightly higher cumulative drug release of EMD NSs at pH 6.5 than that at pH 7.4 might be ascribed to the gradual protonation of DOX under the acidic condition. Based on the overexpressed chemical entities in the cancer TME, such as intracellular H_2_O_2_ and GSH, the above H_2_O_2_-/GSH-responsive characters of EMD NSs are very important in selective cancer therapy.

### 2.5. Different Effects of EMD NSs on Cancer/Normal Cells

**Figure 5:**
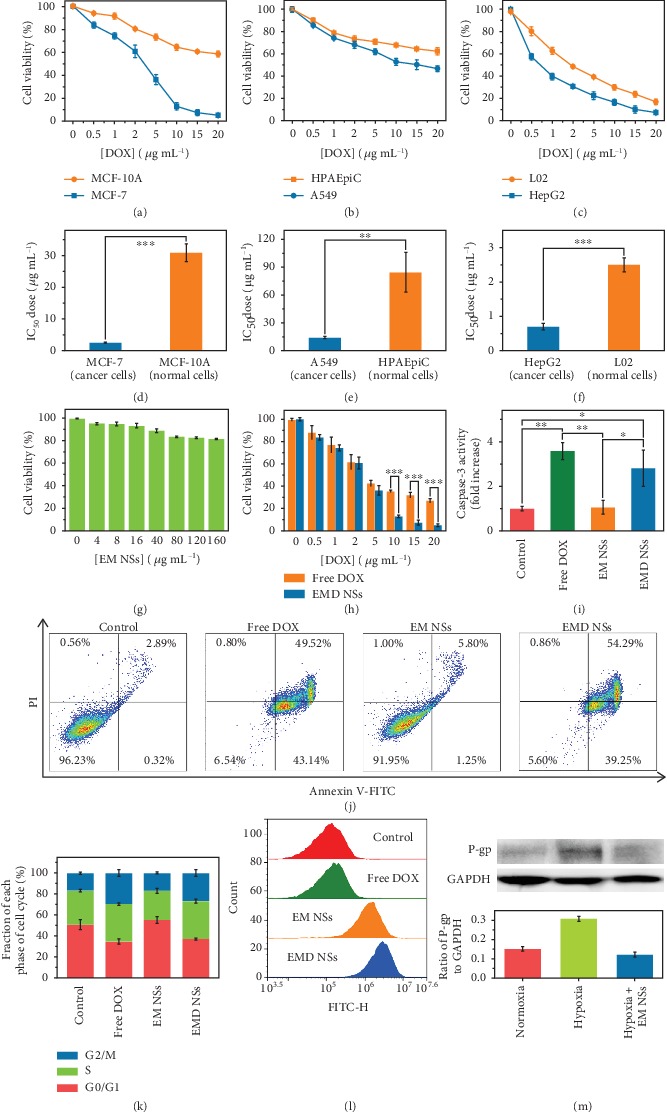
Dose-dependent growth inhibition curves of (a) MCF-7/MCF-10A, (b) A549/HPAEpiC, and (c) HepG2/L02 cells after exposure to EMD NSs at varied DOX concentrations for 24 h. (d–f) Corresponding IC_50_ values of EMD NSs for the above three pairs of cancer/normal cells. (g) Viabilities of MCF-7 cells treated with varied concentrations of EM NSs. (h) Concentration-dependent cytotoxicities of free DOX and EMD NSs against MCF-7 cells. DOX and EM NS concentrations were abbreviated as [DOX] and [EM NSs], respectively, in (a–c, g, and h). Effects of free DOX, EM NSs, and EMD NSs on (i) caspase-3 level, (j) apoptosis/necrosis, and (k) cell cycle distribution of MCF-7 cells. (l) Flow cytometry analysis results of cellular ROS levels after various treatments as indicated. The cells without any treatment were set as the control group. (m) Images and corresponding statistical histogram of western blot results of P-gp expression (with glyceraldehyde-3-phosphate dehydrogenase (GAPDH) as the loading control) of normoxic cells, hypoxic cells, and hypoxic cells treated with EM NSs for 24 h. ^∗^*P* < 0.05, ^∗∗^*P* < 0.01, and ^∗∗∗^*P* < 0.001.

Considering that the GSH concentration in cancer cells is much higher than that in normal cells [30, 31], which was also verified by our cellular total GSH assay results ([Supplementary-material supplementary-material-1]), we would like to investigate whether the EMD NSs respond to the GSH in the two types of cells differently. In the cancer (human breast cancer MCF-7)/normal (human normal mammary epithelial MCF-10A) cell experiments (Figures 4(g) and 4(h)), it can be seen that after treatment with EMD NSs, stronger FL signals were observed in the cell nuclei of MCF-7 cells compared with those in the cell nuclei of MCF-10A cells. These results revealed the excellent cancer/normal cell discrimination ability of EMD NSs, which mainly resulted from their GSH-responsive DOX release. Based on the different DOX release behaviors of EMD NSs in cancer/normal cells, we further assessed the anticancer efficacy of EMD NSs towards three different cancer/normal cell groups by a 3-(4,5-dimethylthiazol-2-thiazolyl)-2,5-diphenyl-2*H*-tetrazolium bromide (MTT) assay. As shown in Figure 5(a), EMD NSs exhibited a stronger inhibitory/killing effect towards cancerous MCF-7 cells than normal MCF-10A cells. According to the dose-dependent cell growth curves, the half maximal inhibitory concentration (IC_50_) value of EMD NSs for MCF-7 cells was calculated to be 2.6 ± 0.1 *μ*g mL^−1^, which was much smaller than that (30.9 ± 2.8 *μ*g mL^−1^) for MCF-10A cells (Figure 5(d)). Additionally, we also observed the stronger cancer cell inhibitory/killing effects in two other pairs of cancer/normal cells (Figures 5(b) and 5(c)), and the differences were quantified by the IC_50_ values of EMD NSs: human lung cancer A549 cells: 14.1 ± 1.2 *μ*g mL^−1^, normal human pulmonary alveolar epithelial HPAEpiC cells: 84.7 ± 21.4 *μ*g mL^−1^, human liver cancer HepG2 cells: 0.7 ± 0.1 *μ*g mL^−1^, and human normal liver L02 cells: 2.5 ± 0.2 *μ*g mL^−1^ (Figures 5(e) and 5(f)).

### 2.6. *In Vitro* Anticancer Treatment and Mechanism

On the basis of the above results, we eventually selected MCF-7 cells for our subsequent experiments to study the anticancer effects of EMD NSs. First, the cytotoxicity of the TME-responsive nanocarriers, EM NSs, was proven to be low by the MTT assay (Figure 5(g)). The viability of MCF-7 cells was concentration-dependent and remained >80% in the EM NS concentration range of 0–160 *μ*g mL^−1^. Importantly, using a total GSH detection kit, it was observed that the total GSH content decreased from 1.75 mM in the cells without treatment ([Supplementary-material supplementary-material-1]) to 0.58 mM in the EM NS-treated cells, suggesting the GSH-depleting ability of EM NSs to enhance the CDT efficiency. Next, at low DOX concentrations (0–5 *μ*g mL^−1^), EMD NSs exhibited the similar cytotoxicity against MCF-7 cells compared with free DOX (Figure 5(h)). We also carried out additional experiments to explore the anticancer mechanisms of EMD NSs. After the MCF-7 cells were treated with free DOX, EM NSs, or EMD NSs for 24 h, the caspase-3 levels are shown in Figure 5(i). The results showed that the apoptosis level induced by EMD NSs was higher than that in the control group and close to that in the “free DOX” group at the same DOX concentration. According to the cell apoptosis detection results (Figure 5(j)), compared with the control group, the cells treated with EMD NSs showed significant early apoptosis (39.25%) and late apoptosis/necrosis (54.29%), which were similar to those treated with free DOX (early apoptosis: 43.14%; late apoptosis/necrosis: 49.52%). Additionally, the evident increase of G2/M phase in cell cycle distribution was observed in both “free DOX” and “EMD NSs” groups (Figure 5(k)). Therefore, the anticancer effect of EMD NSs mainly resulted from the released DOX molecules (evidenced by the confocal imaging results in Figure 4(g)) in this TME-responsive nanosystem. However, at high DOX concentrations (10–20 *μ*g mL^−1^), EMD NSs showed the relatively higher cytotoxicity than free DOX (Figure 5(h)), so we assumed that some other factors may also play roles in the EMD NS-induced cell death. Although EM NSs caused negligible changes in the cell viabilities, the substantial accumulation of ROS was observed in the EM NS- or EMD NS-treated cells (Figure 5(l) and [Supplementary-material supplementary-material-1]), which could be attributed to the conversion of endogenous H_2_O_2_ to highly reactive ^·^OH (one of the most harmful forms of ROS) induced by the Mn^2+^-mediated Fenton-like reaction [58]. Importantly, based on the in vitro EM NS-assisted O_2_ generation results (Figure 4(a)), EM NSs showed great promise in downregulating the P-gp expression by increasing the O_2_ level under hypoxia. Therefore, P-gp expression was also measured by western blot analysis after different treatments. As shown in Figure 5(m), the untreated hypoxic MCF-7 cells exhibited high P-gp expression, which could promote the efflux of DOX. After 24 h incubation of EM NSs, the P-gp expression in hypoxic cells decreased to a relatively low level similar to the expression level in normoxic cells, which indicated that the EM NS-based nanoplatform could inhibit P-gp expression under hypoxia, possessing the potential to reduce drug efflux and thus overcome hypoxia-induced chemoresistance. Taken together, all these observations proved that EMD NSs can efficiently kill cancer cells by the GSH consumption- and O_2_ generation-enhanced chemo-chemodynamic combination therapy.

### 2.7. *In Vivo* Tumor Imaging and Chemo-Chemodynamic Cancer Therapy

**Figure 6:**
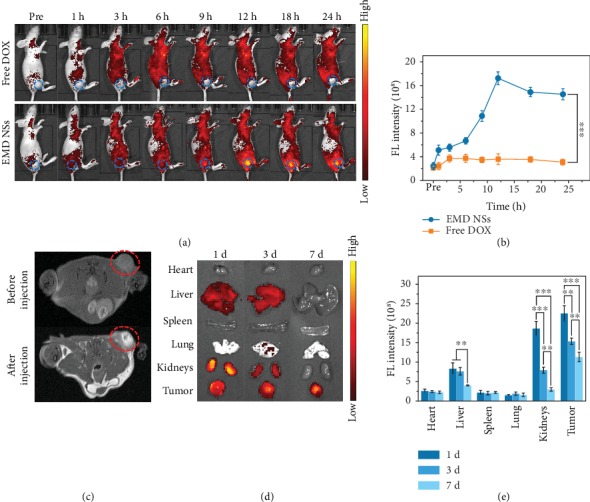
(a) In vivo FL images of tumor-bearing mice before (“Pre”) and after i.v. injection of free DOX or EMD NSs (DOX concentration: 2 mg kg^−1^) for different time periods. The blue dotted circles mark the tumor regions. (b) Corresponding FL intensities in the tumor regions in (a). (c) In vivo MR images of a tumor-bearing mouse before and after i.v. injection of EMD NSs (DOX concentration: 2 mg kg^−1^) for 12 h. The red dotted circles mark the tumor regions. (d) Ex vivo FL images of major organs and tumor tissues of mice sacrificed at 1, 3, and 7 d post i.v. injection of EMD NSs (DOX concentration: 2 mg kg^−1^). (e) Corresponding FL intensities in organs or tissues in (d). ^∗∗^*P* < 0.01 and ^∗∗∗^*P* < 0.001.

Motivated by the in vitro results, we then performed in vivo theranostic experiments using tumor-bearing mice via the tail vein injection with free DOX or EMD NSs. It was observed that free DOX was distributed nonspecifically and rapidly to various organs and tissues after intravenous (i.v.) administration, leading to a rapid elimination from the circulation (Figure 6(a)). Meanwhile, the FL intensity of EMD NSs in the tumor areas showed a continuous increase and reached the maximum at 12 h postinjection followed by a slight decrease, whereas free DOX exhibited very weak FL signals within the whole observation time period (Figure 6(b)). Such a direct FL comparison verifies the excellent tumor targeting ability of EMD NSs, which can be attributed to their proper size (~14 nm in length) that endows them with excellent passive tumor targeting ability via the enhanced permeability and retention effect. Importantly, since the tumor tissues show at least 4-fold higher concentrations of GSH levels compared with normal tissues in the tumor-bearing mice [70], the GSH-triggered “off–on” DOX release of EMD NSs was demonstrated in vivo. As shown in [Supplementary-material supplementary-material-1], a healthy tissue and a tumor tissue in a mouse were injected with EMD NSs, respectively. The negligible FL intensity in the normal tissue and the strong FL intensity in the tumor region provided a solid proof for the selective tumor imaging capability of the nanoagents. Apart from the in vivo FL imaging, the MR images of a tumor-bearing mouse were also acquired before and after the i.v. injection of EMD NSs. As shown in Figure 6(c), a detectable *T*_1_-weighted contrast enhancement was observed in the tumor area at 12 h post i.v. injection, which proved that the obtained EMD NSs can be used as promising contrast agents for MR imaging application. In addition, the mice intravenously injected with EMD NSs were sacrificed at 1, 3, and 7 d postinjection, respectively. Ex vivo images and quantified FL intensities revealed that EMD NSs were distributed mainly in tumors and partially in livers and kidneys at 1 and 3 d postinjection and were still retained in the tumor at 7 d postinjection without evident distributions in other organs (Figures 6(d) and 6(e)), which indicated their excellent tumor accumulation, prolonged tumor retention, and good biosafety. Besides, the FL intensities in the kidneys were higher than those in the livers, implying that EMD NSs might be mainly captured and metabolized by kidneys due to their small size.

**Figure 7:**
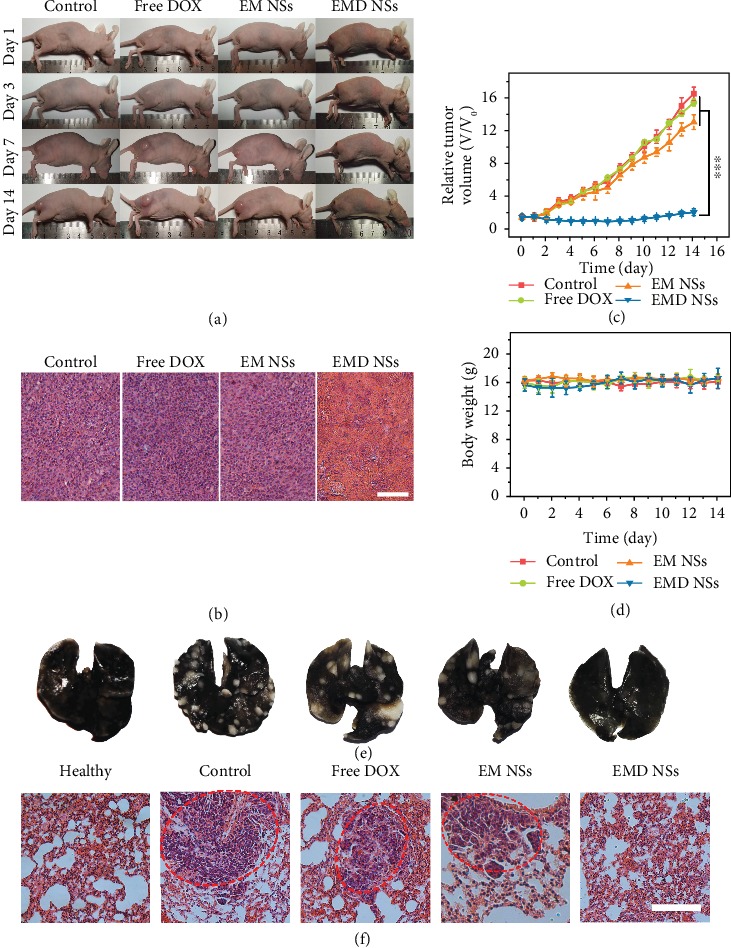
(a) Photographs of differently treated mice taken at 1, 3, 7, and 14 d. (b) H&E-stained slices of tumor issues taken on the 14th day. Scale bar = 100 *μ*m. (c) Relative tumor growth curves and (d) body weight curves in the three groups. ^∗∗∗^*P* < 0.001. (e) Photographs of India ink-filled whole lungs from mice in various groups and (f) corresponding H&E-stained lung slices. The red dashed circles mark the tumor metastasis sites. Scale bar = 100 *μ*m.

Subsequently, inspired by the high tumor accumulation of EMD NSs, the in vivo tumor suppression activity of the TME-responsive nanoagents was evaluated using tumor-bearing mice. As shown in Figures 7(a) and 7(c), the tumor growth of the mice intravenously injected with EMD NSs was almost completely suppressed during the period of our observation (14 days), whereas free DOX did not exhibit noticeable therapeutic efficacy. Additionally, the tumor growth of mice treated with EM NSs was also not effectively suppressed, which might be due to the inadequate administration of EM NSs. The hematoxylin and eosin- (H&E-) stained tumor slices from the EMD NS-treated group showed that most cells were severely damaged after treatment (Figure 7(b)), further confirming the satisfactory in vivo therapeutic outcome of the nanoagents. Furthermore, the tumor lung metastasis was examined on the 30th day after various treatments. As revealed by the photographs of India ink-stained whole lungs and the H&E-stained lung slices (Figures 7(e) and 7(f)), a large number of tumor nodules were observed in the lungs of untreated, DOX-treated, and EM NS-treated tumor-bearing mice, while no obvious lung metastasis was observed in the EMD NS-treated group, suggesting the excellent inhibitory effect of EMD NSs on tumor metastasis.

We also investigated the potential side effects of EMD NSs. First, no evident body weight loss was observed during the treatments (Figure 7(d)), suggesting the negligible toxic effect of EMD NSs on the animals. Next, according to the tissue slices from the EMD NS-treated mice at 14 d postinjection ([Supplementary-material supplementary-material-1]), compared with the control group, no obvious cell apoptosis/necrosis was observed in the major organs, indicating the good biosafety of the nanoagents, which may result from the efficient excretion of the nanoagents by the mice. Besides, even at the high DOX concentration of 200 *μ*g mL^−1^, EMD NSs did not induce hemolysis (Figures [Supplementary-material supplementary-material-1] and [Supplementary-material supplementary-material-1]), and no abnormalities in the detected blood indices were seen in the blood routine analysis for the mice sacrificed on the 14th day after EMD NS treatment (Figures [Supplementary-material supplementary-material-1]), showing the good hemocompatibility of EMD NSs. Collectively, the above results demonstrated that EMD NSs can be used as safe antitumor nanoagents.

## 3. Conclusion

In summary, we have developed a TME-responsive, bacterium-derived, and MnO_*x*_-based nanoplatform which can efficiently load the anticancer drug DOX for GSH detection as well as chemo-chemodynamic combination therapy. Interestingly, during the synthesis of the MnO_*x*_-based smart drug carrier, the *E. coli* bacterial cells were used as both the template and the reducing agent. The as-obtained nanocarriers exhibit a spindle-like morphology with an average size of 13.4 × 1.7 nm, good colloidal stability, and multiple responses. The DOX-loaded bacterium/MnO_*x*_-based nanospindles with high drug encapsulation efficiency (~100.0%) and drug loading efficiency (11.1%) show the following advantages: (1) The loaded DOX can be used not only as an anticancer drug but also as a fluorescent probe. Based on the FL quenching of DOX by EM NSs and the FL recovery triggered by GSH, EMD NSs can serve as a selective and sensitive GSH sensor. (2) The GSH-triggered DOX release and Mn^2+^ production can realize the FL and *T*_1_-weighted MR imaging, respectively, which is highly beneficial for the accuracy and effectiveness of cancer treatment. (3) EMD NSs exhibit both Fenton-like Mn^2+^ delivery and GSH depletion properties for enhanced CDT. (4) The in situ generated O_2_ resulting from EM NS-triggered decomposition of tumor endogenous H_2_O_2_ can inhibit the expression of P-gp, which is crucial for overcoming hypoxia-associated chemoresistance. (5) Since the DOX molecule can be replaced by other small molecule drugs (such as photosensitizers, photothermal molecules, and other chemotherapeutics), the MnO_*x*_-based nanoplatform holds great potential for universal drug delivery and various disease treatments. Importantly, the present work develops a facile bacterium-based biosynthetic strategy to construct smart drug nanocarriers for loading chemotherapeutics and realizing chemo-chemodynamic combination therapy, which we believe may find applications in research areas such as nanomedicine and biosensing.

## 4. Materials and Methods

### 4.1. Materials

*E. coli* and *S*. *aureus* were purchased from China Center of Industrial Culture Collection (CICC, Beijing, China). KMnO_4_ was purchased from Chengdu Kelong Chemical Inc. (Chengdu, China). Doxorubicin hydrochloride (DOX) was purchased from Beijing Huafeng United Technology Co., Ltd. (China). L-Glutathione (GSH) was purchased from Sigma-Aldrich (St. Louis, MO). Dialysis membranes (Spectra/Por® 6, regenerated cellulose) with the MWCO of 10 kDa were purchased from SpectrumLabs, Inc. (Rancho Dominguez, CA). All solutions/suspensions were prepared with deionized (DI) water (18.2 M*Ω*·cm) purified by a Milli-Q system (Millipore, Billerica, MA). The Annexin V-Fluorescein Isothiocyanate (FITC) Apoptosis Detection Kit containing annexin V/propidium iodide (PI), Caspase-3 Colorimetric Assay Kit, Annexin V-FITC Apoptosis Detection Kit, Cell Cycle Detection Kit, and ROS Detection Kit were all purchased from Nanjing KeyGen Biotech Co. Ltd.

### 4.2. Instruments and Characterization

The TEM experiment was performed using a transmission electron microscope (JEM-2100, JEOL Ltd., Japan). Before measurements, the samples were prepared by dropping the sample suspensions onto the ultrathin carbon-coated copper grids and air-drying. The size distribution was obtained by counting at least 100 particles from one TEM image per sample using an image analysis software (Nano Measurer 1.2). For XRD analysis, the fine EM NS powder was first obtained by freeze-drying and grinding. Then, the powder was placed into the sample holder and gently pressed using a glass slide to make the surface smooth. XRD patterns were then recorded on an XRD system (D8 Discover, Bruker, Germany). The FTIR spectra of the freeze-dried EM NS powder and bacteria (*E. coli*) were collected with a Nicolet iS50 FTIR spectrometer (Thermo Fisher Scientific Co., Waltham, MA, USA). Additionally, the EM NS powder was sprinkled onto the surface of sticky carbon conductive tape for XPS analysis using an X-ray photoelectron spectrometer (Quantera 2000, Ulvac-Phi Inc., Japan). UV–vis spectra were obtained using a UV–vis spectrophotometer (UV-2600, Shimadzu, Japan). The FL spectra were obtained on a spectrophotometer (RF-5301PC, Shimadzu, Japan). The hydrodynamic diameter and zeta potential measurements were conducted on a Zetasizer instrument (Nano ZS, Malvern Instruments, UK). To determine the Mn concentration in the EM NS suspension, the sample was first digested with aqua regia at 60°C overnight and then diluted with DI water for analysis using ICP-OES (Perkin-Elmer Optima 2100DV, USA).

### 4.3. Animal Model

Female BALB/c nude mice were obtained from Yangzhou University Medical Center (Yangzhou, China) and used under protocols approved by the Southeast University Laboratory Animal Center. 100 *μ*L of PBS containing 4T1 cells (2 × 10^6^) was subcutaneously inoculated onto the back of each mouse to build the xenograft tumor model.

## Supplementary Materials

Supplementary Materials Table S1: GSH levels in various cancer/normal cells. Figure S1: enlarged version of Figure 2(a) for clearer observation. Figure S2: size distribution of EM NSs dispersed in PBS as measured by DLS. Figure S3: TEM images of the suspensions obtained by (a) *B. subtilis* and (b) *S. aureus* cells treated with KMnO_4_ and ultrasonication. TEM images of the suspensions obtained by (c) *B. subtilis* and (d) *S. aureus* cells that were first preincubated with 1% Triton X-100 solution overnight and then treated with KMnO_4_ and ultrasonication. Figure S4: FTIR spectrum of the freeze-dried *E. coli* bacteria. Figure S5: XPS high-resolution (a) O 1s, (b) N 1s, and (c) Mn 2p curves. Figure S6: hydrodynamic size changes of EM NSs dispersed in PBS or DMEM for a duration of two weeks as measured by DLS. Figure S7: enlarged version of Figure 3(a) for clearer observation. Figure S8: UV–vis absorption spectra of aqueous solutions containing free DOX, EM NSs, EMD NSs, or EMD NSs+GSH. Figure S9: FL emission spectra of aqueous solutions containing free DOX, EM NSs, EMD NSs, or EMD NSs + GSH. Ex = 500 nm. Figure S10: TEM image (left) and corresponding size histogram (right) of EM NSs after treatment with GSH (2.0 mM). Figure S11: photographs of EMD NS suspensions after mixing with various concentrations of GSH under a UV lamp (365 nm excitation). The final GSH concentrations are indicated in the figure. Figure S12: FL responses of EMD NSs to different guest molecules. The concentrations of all guests were 1.0 mM. Figure S13: flow cytometry-based FL analysis of cellular ROS levels after various treatments as indicated. ^∗∗∗^*P* < 0.001. Figure S14: in vivo FL image (inset) and corresponding FL analysis result of a mouse injected with EMD NSs. “1” and “2” indicate the normal and tumor areas injected with EMD NSs, respectively. ^∗∗^*P* < 0.01. Figure S15: histological analysis of major organs collected from mice sacrificed on the 14th day post i.v. injection with PBS (control), free DOX, EM NSs, or EMD NSs. Scale bar = 100 *μ*m. Figure S16: (a) photographs of RBC suspensions after hemolysis assay. (b) Hemolysis percentages of RBCs treated with PBS (negative control) and Triton X-100 (positive control), or varied concentrations of free DOX or EMD NSs. DOX concentration was abbreviated as [DOX] in (a and b). (c–n) Blood routine analysis results of mice after treatment with PBS (control), free DOX, or EMD NSs. The blood indexes including WBC, LYM, MONO, NEUT, RBC, HGB, HCT, MCV, MCH, MCHC, PLT, and MPV indicate numbers of white blood cells, lymphocytes, monocytes, neutrophilic granulocytes, red blood cells, concentration of hemoglobin, hematocrit, mean corpuscular volume, mean corpuscular hemoglobin, mean corpuscular hemoglobin concentration, platelet count, and mean platelet volume, respectively.
