# Supplementary material for: Bacterial Template Synthesis of Multifunctional Nanospindles for Glutathione Detection and Enhanced Cancer-Specific Chemo-Chemodynamic Therapy
Source: Research (Wash D C). 2020 Mar 26;2020:9301215. doi: 10.34133/2020/9301215 (PMC7136754; doi:10.34133/2020/9301215)
Supplement: Supplementary Materials — Table S1: GSH levels in various cancer/normal cells. Figure S1: enlarged version of Figure 2(a) for clearer observation. Figure S2: size distribution of EM NSs dispersed in PBS as measured by DLS. Figure S3: TEM images of the suspensions obtained by (a) B. subtilis and (b) S. aureus cells treated with KMnO4 and ultrasonication. TEM images of the suspensions obtained by (c) B. subtilis and (d) S. aureus cells that were first preincubated with 1% Triton X-100 solution overnight and then treated with KMnO4 and ultrasonication. Figure S4: FTIR spectrum of the freeze-dried E. coli bacteria. Figure S5: XPS high-resolution (a) O 1s, (b) N 1s, and (c) Mn 2p curves. Figure S6: hydrodynamic size changes of EM NSs dispersed in PBS or DMEM for a duration of two weeks as measured by DLS. Figure S7: enlarged version of Figure 3(a) for clearer observation. Figure S8: UV–vis absorption spectra of aqueous solutions containing free DOX, EM NSs, EMD NSs, or EMD NSs+GSH. Figure S9: FL emission spectra of aqueous solutions containing free DOX, EM NSs, EMD NSs, or EMD NSs + GSH. Ex = 500 nm. Figure S10: TEM image (left) and corresponding size histogram (right) of EM NSs after treatment with GSH (2.0 mM). Figure S11: photographs of EMD NS suspensions after mixing with various concentrations of GSH under a UV lamp (365 nm excitation). The final GSH concentrations are indicated in the figure. Figure S12: FL responses of EMD NSs to different guest molecules. The concentrations of all guests were 1.0 mM. Figure S13: flow cytometry-based FL analysis of cellular ROS levels after various treatments as indicated. ∗∗∗P < 0.001. Figure S14: in vivo FL image (inset) and corresponding FL analysis result of a mouse injected with EMD NSs. “1” and “2” indicate the normal and tumor areas injected with EMD NSs, respectively. ∗∗P < 0.01. Figure S15: histological analysis of major organs collected from mice sacrificed on the 14th day post i.v. injection with PBS (control), free DOX, EM NSs, or EMD NSs. Scal [file 9301215.f1.doc]

**Supplementary Materials**

**Bacterial Template Synthesis of Multifunctional Nanospindles for Glutathione Detection and Enhanced Cancer-Specific Chemo-Chemodynamic Therapy**

Yan-Wen Bao, Xian-Wu Hua, Jia Zeng, Fu-Gen Wu*

State Key Laboratory of Bioelectronics, School of Biological Science and Medical Engineering, Southeast University, 2 Sipailou Road, Nanjing 210096, P. R. China

* Correspondence should be addressed to Fu-Gen Wu; wufg@seu.edu.cn

**1. Experimental section**

**1.1. Synthesis of EM NSs**

First, *E. coli* was cultured in lysogeny broth (LB) medium on a shaking incubator (200 rpm) at 37 °C. After culturing for 24 h, 1 mL of *E. coli* suspension was centrifuged (10 krpm, 15 min) to remove culture medium, and then cells were washed with deionized (DI) water three times through centrifugation and resuspended in DI water (3 mL). Thereafter, 60 μL of KMnO_4_ solution (20 mg mL^−1^) was added and the mixture was tip-sonicated by an ultrasonic cell crusher (XO-650D, Nanjing Xianou Instrument Manufacturing Co., Ltd., Nanjing, China) for 15 min at 35% of the power output. The brown EM NS suspension was purified by dialysis (molecular weight cut-off (MWCO): 10 kDa) and centrifugation (10 krpm, 15 min). The concentration of the resultant EM NS suspension was determined by weighing the dry powder obtained by freeze-drying of a portion of the suspension. The remaining EM NS suspension was diluted to 1 mg mL^−1^ and stored at 4°C for further experiments. For comparison, *E. coli* was replaced by *B*. *subtilis* or *S*. *aureus* (without or with 24-h pre-incubation of 1% Triton X-100 solution) during the same KMnO_4_ and ultrasonication treatments. Then, the obtained suspensions were imaged by TEM. To investigate the colloidal stability, the size distributions of EM NSs dispersed in PBS and DMEM were measured by DLS every other day for two weeks, respectively.

**1.2. Synthesis of EMD NSs**

For preparation of EMD NSs, 50 μL of DOX solution (2 mg mL^−1^) was added to 800 μL of EM NS suspension (1 mg mL^−1^). The mixture was vortexed for 1 min. After centrifugation at 15k rpm for 30 min, the DOX encapsulation efficiency and loading efficiency were calculated by the following equations: Encapsulation efficiency = (1 – weight of unencapsulated Dox/total weight of DOX fed initially) × 100%; Loading efficiency = (total of DOX fed initially – weight of unencapsulated DOX)/total weight of EMD NSs × 100%.

**1.3. GSH detection assay**

The EMD NSs (DOX concentration: 10 µg mL^−1^) was incubated with varied concentrations (0, 1, 2, 5, 10, 20, 30, 50, 70, 100, 150, 200, 300, 400, 500, 700, 1000, 1200, 1500, and 2000 µM) of GSH in PBS for 30 min at room temperature before collecting the FL spectra. Each sample was excited at 500 nm, while the emission wavelength was collected from 510 to 690 nm. All the above concentrations were the final concentrations.

**1.4. O_2_ generation assay**

Three groups of solutions were prepared in water: (1) H_2_O_2_ (100 μM), (2) EM NSs (100 μg mL^−1^) + H_2_O_2_ (100 μM), and (3) EMD NSs (EM NS concentration: 100 μg mL^−1^) + H_2_O_2_ (100 μM). The dissolved O_2_ in different solutions was tested using a portable dissolved oxygen meter (JPB-607A, Shanghai Precision and Scientific Instrument Co., Ltd., China).

**1.5. •OH generation assay**

Seven groups of solutions were prepared: (1) MB (H_2_O), (2) MB + H_2_O_2_, (3) MB + H_2_O_2_ + Mn^2+^ (H_2_O), (4) MB + H_2_O_2_ + Mn^2+^, (5) MB + H_2_O_2_ + Mn^2+^ + GSH, (6) MB + H_2_O_2_ + EM NSs, and (7) MB + H_2_O_2_ + EM NSs + GSH. The concentrations of MB, H_2_O_2_, Mn^2+^, GSH, and EM NSs were 10 μg mL^−1^, 8 mM, 0.5 mM, 2.0 mM, and 1 mg mL^−1^, respectively. The groups 1 and 3 were prepared in H_2_O, and the groups 2 and 4–7 were prepared in 25 mM CO_3_^2−^/HCO_3_^−^ buffer solution. Especially, the EM NSs in the group 7 were pre-incubated at 37°C for 15 min. All the solutions were incubated at 37°C for 30 min, photographed, and measured by UV–vis spectroscopy.

**1.6. DOX release from EMD NSs**

The EMD NS suspension (DOX concentration: 100 μg mL^−1^, 1.5 mL) in a dialysis membrane (MWCO: 10 kDa) was dispersed in PBS (pH = 6.5 or 7.4, 15 mL) with or without GSH (2.0 mM) for 24 h at 37°C. At various time points (0, 1, 2, 3, 4, 6, 8, 10, 12, 19, and 24 h), the solution outside the dialysis membrane was measured using the UV–vis spectrophotometer to detect the released DOX.

**1.7. Cell culture**

MCF-7, A549, HPAEpiC, HepG2, L02, and murine breast cancer 4T1 cells were grown with DMEM supplemented with 10% fetal bovine serum (FBS), 100 U mL^−1^ of penicillin, and 100 μg mL^−1^ of streptomycin. MCF-10A cells were cultured in endothelial cell medium (ECM) containing 5% FBS, 1× endothelial cell growth supplement (ECGS), 100 U mL^−1^ of penicillin, and 100 µg mL^−1^ of streptomycin. All cells were cultured in a humidified incubator at 37°C and 5% CO_2_.

**1.8. Cellular experiments**

In cell experiments, the cells were observed under a confocal laser scanning microscope (TCS SP8, Leica, Germany) and the cellular FL intensity was quantified by a flow cytometer (NovoCyte 2060, ACEA Bioscience, USA). Additionally, cells were first treated without or with EM NSs (40 μg mL^–1^) in cell culture flasks for 24 h and then the total GSH contents were detected using a total GSH detection kit (Beyotime, China).

For cell imaging, MCF-7 or MCF-10A cells were pre-seeded in an 8-well plate. After culturing for 24 h, the culture medium was replaced by 200 μL of fresh medium containing free DOX (5 μg mL^−1^), EM NSs (40 μg mL^−1^), or EMD NSs (DOX concentration: 5 μg mL^−1^). After incubation for another 24 h, the cells were stained with Hoechst 33342 for 10 min and analyzed by confocal imaging and flow cytometry. The cells without drug treatment were set as the control group.

For in vitro cytotoxicity test, cells were pre-seeded at 5 × 10^3^ cells/well in 96-well plates and cultured for 24 h. Subsequently, cells were incubated with the fresh medium containing EM NSs or EMD NSs at varied concentrations for another 24 h. A standard MTT assay was carried out to determine the relative cell viabilities. Three replicates were done for each group. The half maximal inhibitory concentration (IC_50_) values for different cell lines were calculated using GraphPad Prism.

To investigate the anticancer effects of EMD NSs, cells were pre-seeded in dishes and cultured for 24 h. Then, they were exposed to free DOX (5 μg mL^−1^), EM NSs (40 μg mL^−1^), or EMD NSs (DOX concentration: 5 μg mL^−1^) for another 24 h. The cells without drug treatment were set as the control group. Thereafter, caspase-3 activity measurement, apoptosis/necrosis assay, cell cycle distribution measurement, and ROS detection were conducted according to the protocols of the corresponding assay kits, respectively.

**1.9. Western blot analysis of P-gp**

MCF-7 cells were seeded in cell culture flasks (75 cm^2^) and cultured for 48 h in normoxia or hypoxia. For incubation under the hypoxic condition, cells were cultured with complete medium and incubated in a hypoxia chamber (self-made) with 1% O_2_, 5% CO_2_, and 94% N_2_ at 37°C. Then, one group of hypoxic cells was incubated with EM NSs at 40 μg mL^–1^. After 24 h incubation, three groups of cells (normoxia, hypoxia, and hypoxia + EM NSs) were washed with cold PBS and lysed in CytoBuster Protein Extraction Buffer (Novagen, San Diego) with 1% Protease Inhibitor Cocktail Set III, EDTA-Free (Calbiochem., Germany) on ice. Bicinchoninic acid (BCA) assay was performed to measure the protein concentrations, and equal amounts of proteins were resolved by sodium dodecyl sulfate polyacrylamide gel electrophoresis (SDS-PAGE) and transferred onto polyvinylidene difluoride (PVDF) membranes. Immediately, the blotted membranes were blocked in Tris-buffered saline Tween-20 (TBST) containing 5% nonfat milk for 1.5 h. The antibody to P-gp (1 : 1500, Abway Antibody Technology Co., Ltd., Beijing, China) as the primary antibody was incubated with the membranes overnight at 4°C with shaking. Afterwards, the blots were incubated with horseradish peroxidase-conjugated secondary antibody (1 : 1500) for 1 h. The blots were washed three times with TBST after the incubation of primary and secondary antibodies. Finally, target proteins were detected by enhanced chemiluminescence (ECL) kit (KeyGen Biotech, China) and visualized by chemiluminescence imaging system (Tanon-5200, China).

**1.10. MR imaging**

For in vitro MR imaging, EMD NS suspensions with or without GSH (2.0 mM) at varied Mn concentrations were scanned under a Bruker 7.0 T PharmaScan system (BioSpin MRI GmbH, Germany). After acquiring the *T*_1_-weighted MR images, the signal intensities were measured for each sample in the region of interest (ROI). Relaxation rates *r*_1_ (1/*T*_1_) were calculated from *T*_1_ values at varied Mn concentrations. For in vivo MR imaging, *T*_1_-weighted MR images of tumor-bearing mice before and after i.v. injection with EMD NSs (DOX concentration: 2 mg kg^−1^) for 12 h were also acquired.

**1.11. In vivo and ex vivo FL imaging**

The tumor-bearing mice were intravenously injected with 100 µL of PBS containing free DOX or EMD NSs (DOX concentration: 2 mg kg^–1^). Then the in vivo FL images of treated mice were obtained at different time points (1, 3, 6, 9, 12, 18, and 24 h postinjection) by a PerkinElmer in vivo imaging system (IVIS Lumina XRMS Series III) at an excitation wavelength of 500 nm and an emission wavelength of 620 nm. The in vivo FL images were also taken before injection for comparison purposes. Furthermore, to explore the biodistribution of EMD NSs, at 1, 3, and 7 d postinjection, the major organs (hearts, livers, spleens, lungs, and kidneys) and tumors of treated mice were collected and imaged. All the FL intensities were measured using the PerkinElmer Image Analysis Software.

**1.12. In vivo anticancer treatment and lung metastasis evaluation**

The tumor bearing mice were randomly divided into four groups (three mice per group): the mice were intravenously injected with 100 μL of PBS (control), free DOX, EM NSs, and EMD NSs (DOX concentration: 2 mg kg^–1^), respectively. Tumor volumes and weights of mice were monitored every day for 14 days. Tumor volumes were calculated as width^2^ × length/2. Additionally, 30 days after treatments, the treated mice were sacrificed at desired time points right after India ink (15%) was injected into their lungs through the trachea. The lungs were then collected and soaked in a Fekete’s solution (100 mL of 70% alcohol, 10 mL of formalin, and 5 mL of glacial acetic acid). The metastatic lesions appear as white nodules on the black lung surfaces after this procedure. After being photographed, the lungs were embedded in paraffin, cut into sections, and subjected to H&E staining. For comparison purposes, the photographing and H&E staining assay were also conducted for the lung from a healthy mouse.

**1.13. In vivo toxicity assay**

To investigate the biosafety of free DOX, EM NSs, and EMD NSs, On the one hand, representative mice from different groups (PBS (control), free DOX, EM NSs, and EMD NSs) were sacrificed on the 14th day post treatment and their major organs and tumors were dissected and then fixed in 4% formaldehyde solutions, followed by making paraffin sections. Next, the paraffin sections were stained with H&E and then observed under an optical microscope. On the other hand, the blood samples in the groups of PBS (control), free DOX, and EMD NSs were collected on the 14th day post treatment to conduct blood biochemical assay.

**1.14. Hemolysis assay**

Blood from a healthy mouse was collected in tubes containing heparin sodium and used immediately. RBCs were collected by centrifugation (1 krpm, 5 min), washed with PBS three times, and diluted with PBS. 100 μL of diluted RBC suspension was then mixed with 400 μL of free DOX solution or EMD NS suspension at varied final DOX concentrations (5, 10, 20, 50, 100, and 200 mL^–1^). RBCs incubated with 1% Triton X-100 solution and PBS solution were set as positive and negative control, respectively. All the mixtures were vortexed and kept at room temperature for 2 h. Finally, the mixtures were centrifuged at 5 krpm for 5 min, and the absorbance of the supernatant at 570 nm (*A*) for each sample was determined using a microplate reader (Multiskan FC, Thermo Scientific, USA). The percentage of hemolysis was calculated as follow: Hemolysis (%) = (*A*_sample_ – *A*_negative control_)/(*A*_positive control_ – *A*_negative control_) × 100%.

**1.15. Statistical analysis**

All the statistical data were presented as the mean values ± standard deviations from three parallel experiments per group. Statistical analyses were carried out using one-way analysis of variance (ANOVA) and and *P* value less than 0.05 was considered to be statistically significant (**P* < 0.05, ***P* < 0.01, ****P* < 0.001).

**2. Additional table and figures**

**Table S1.** GSH levels in various cancer/normal cells.

| Cell type | **Cancer cells** | | | **Normal cells** | | |
| --- | --- | --- | --- | --- | --- | --- |
|  | MCF-7 | A549 | HepG2 | MCF-10A | HPAEpiC | L02 |
| GSH level (μM) | 1753.8 | 1223.1 | 1107.7 | 305.1 | 206.1 | 194.5 |


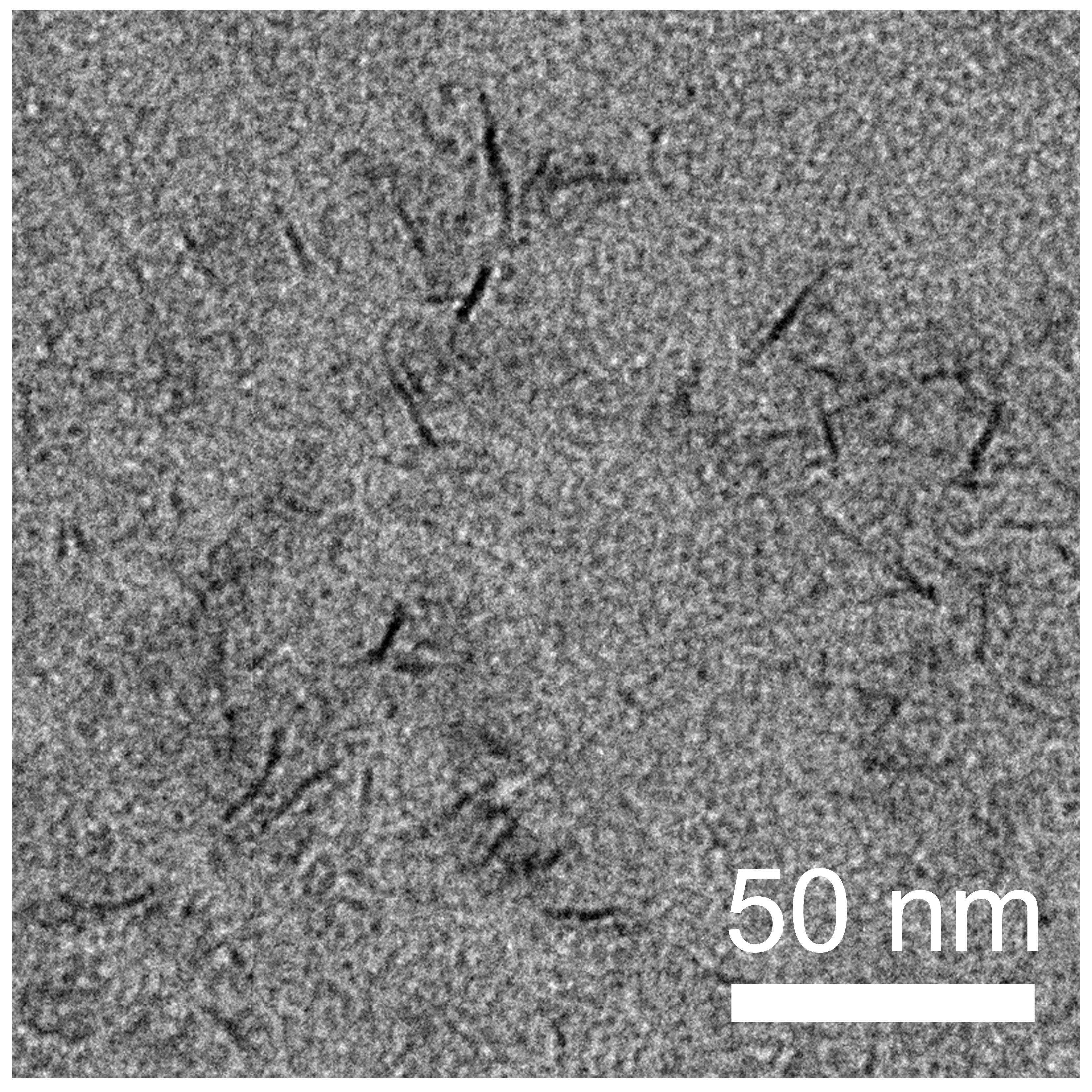


**Figure S1.** Enlarged version of Figure 2(a) for clearer observation.


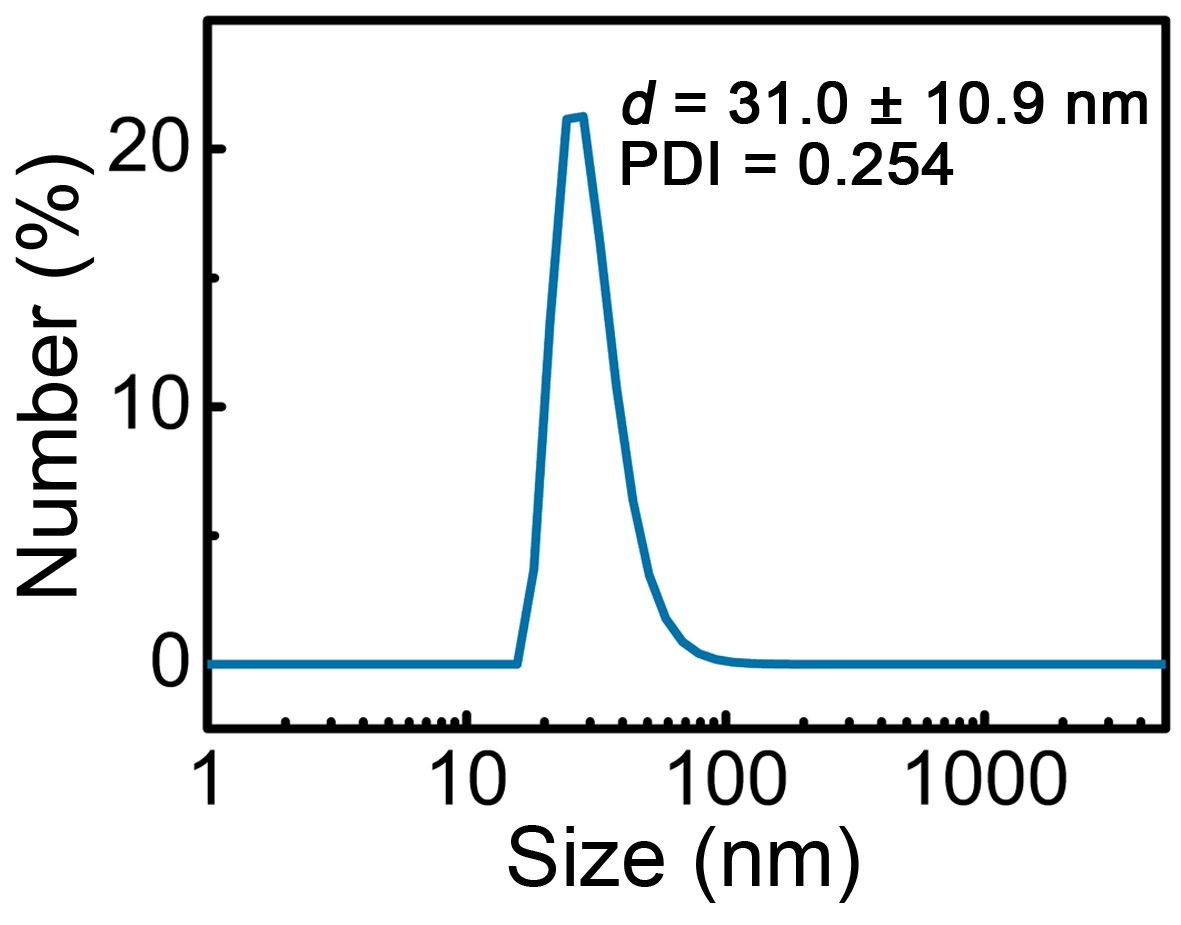


**Figure S2.** Size distribution of EM NSs dispersed in PBS as measured by DLS.


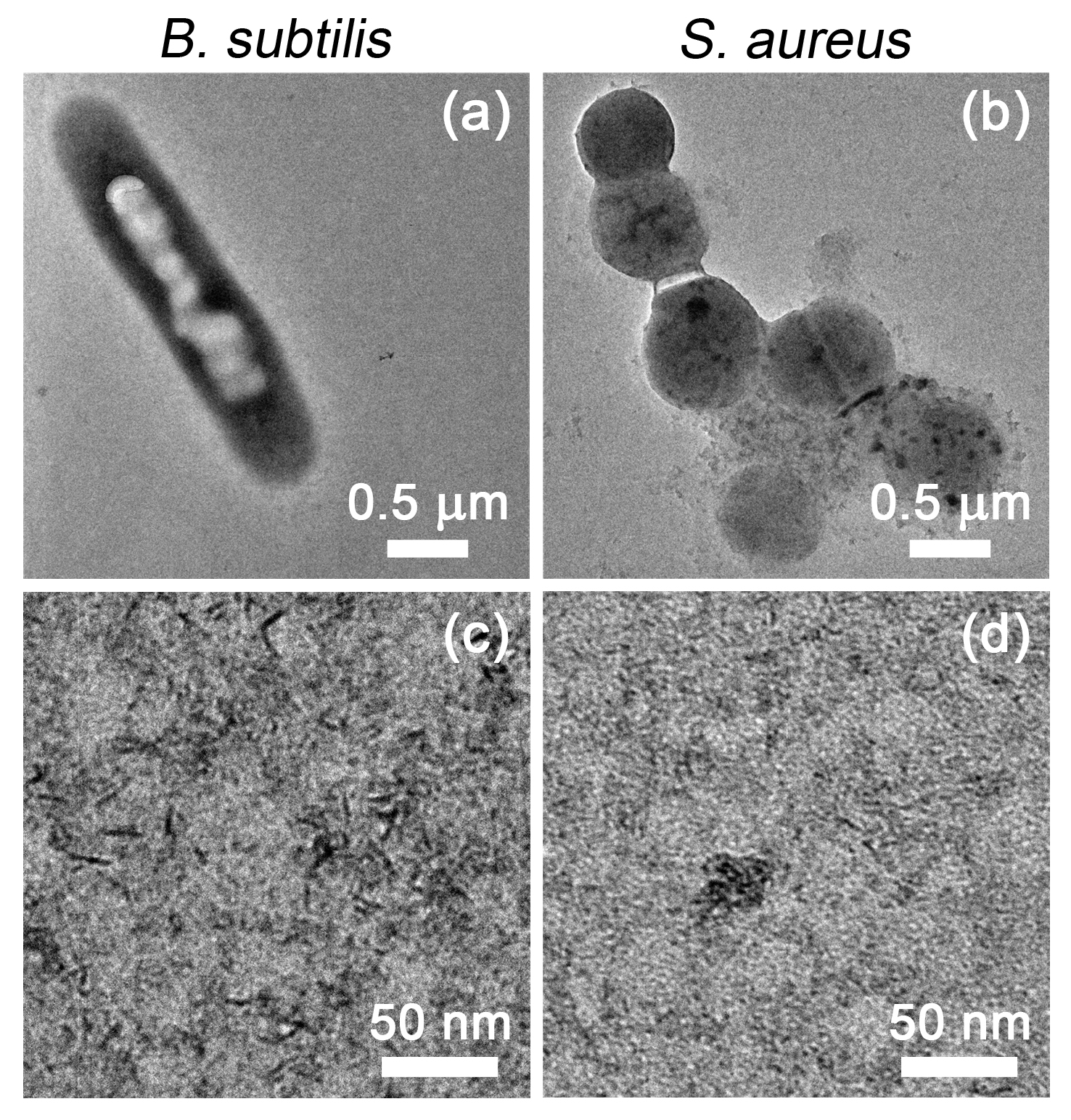


**Figure S3.** TEM images of the suspensions obtained by (a) *B*. *subtilis* and (b) *S. aureus* cells treated with KMnO_4_ and ultrasonication. TEM images of the suspensions obtained by (c) *B*. *subtilis* and (d) *S. aureus* cells that were first pre-incubated with 1% Triton X-100 solution overnight and then treated with KMnO_4_ and ultrasonication.


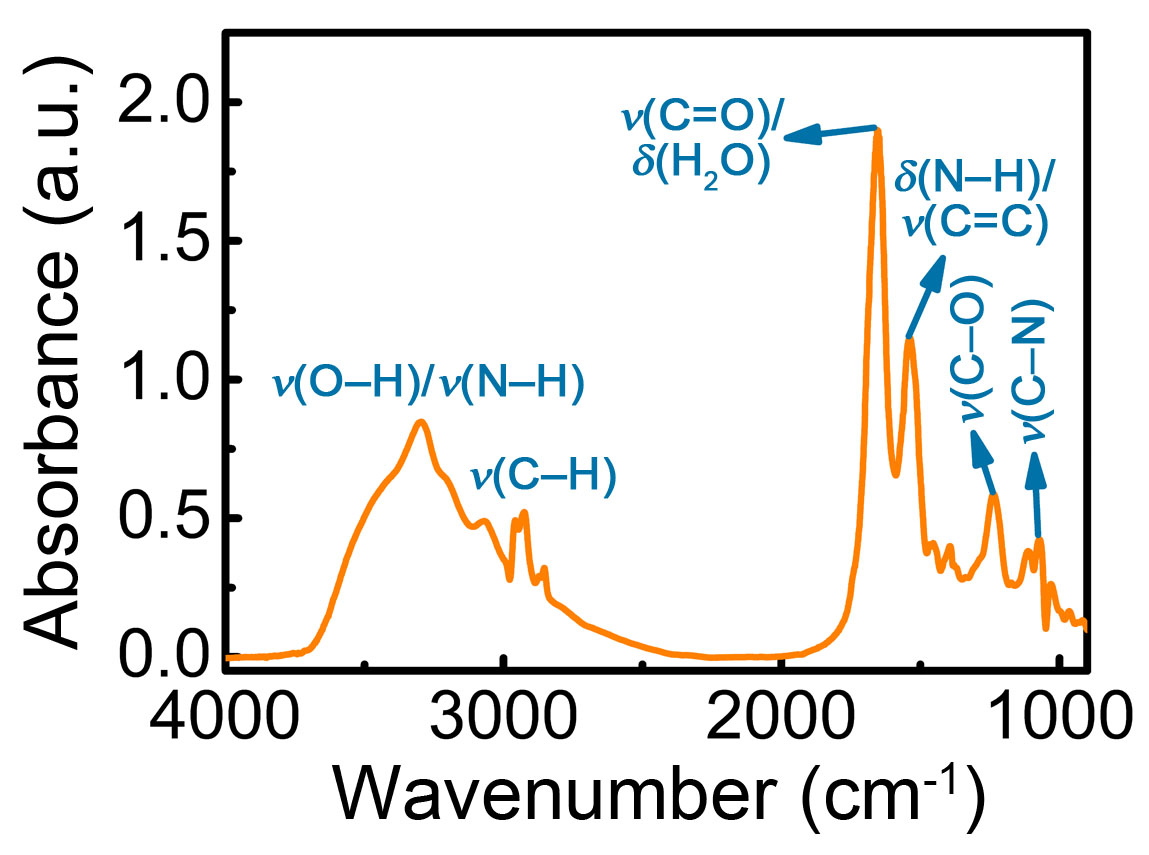


**Figure S4.** FTIR spectrum of the freeze-dried *E. coli* bacteria.


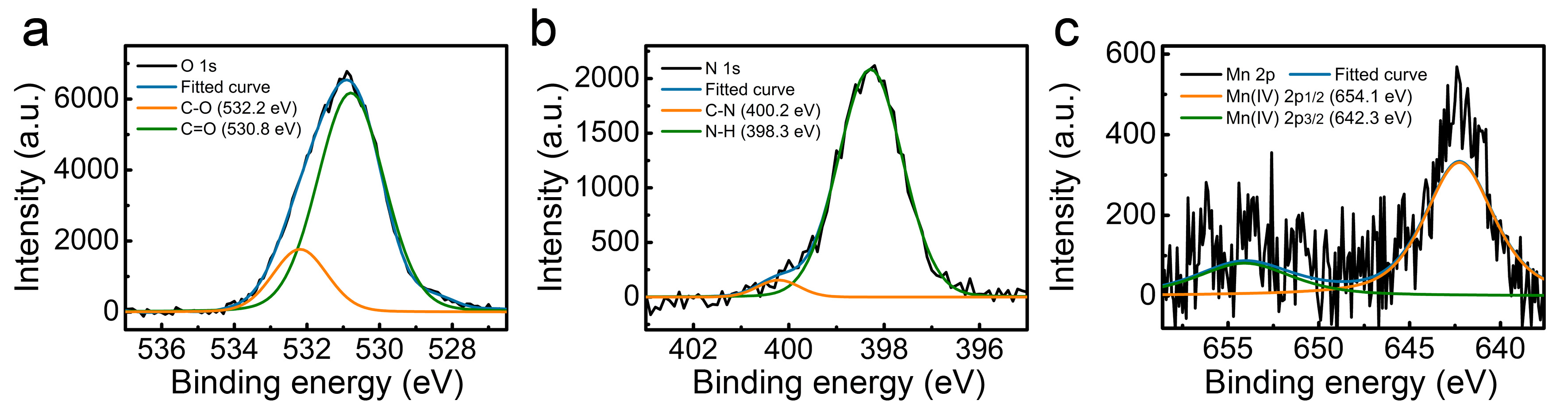


**Figure S5.** XPS high-resolution (a) O 1s, (b) N 1s, and (c) Mn 2p curves.


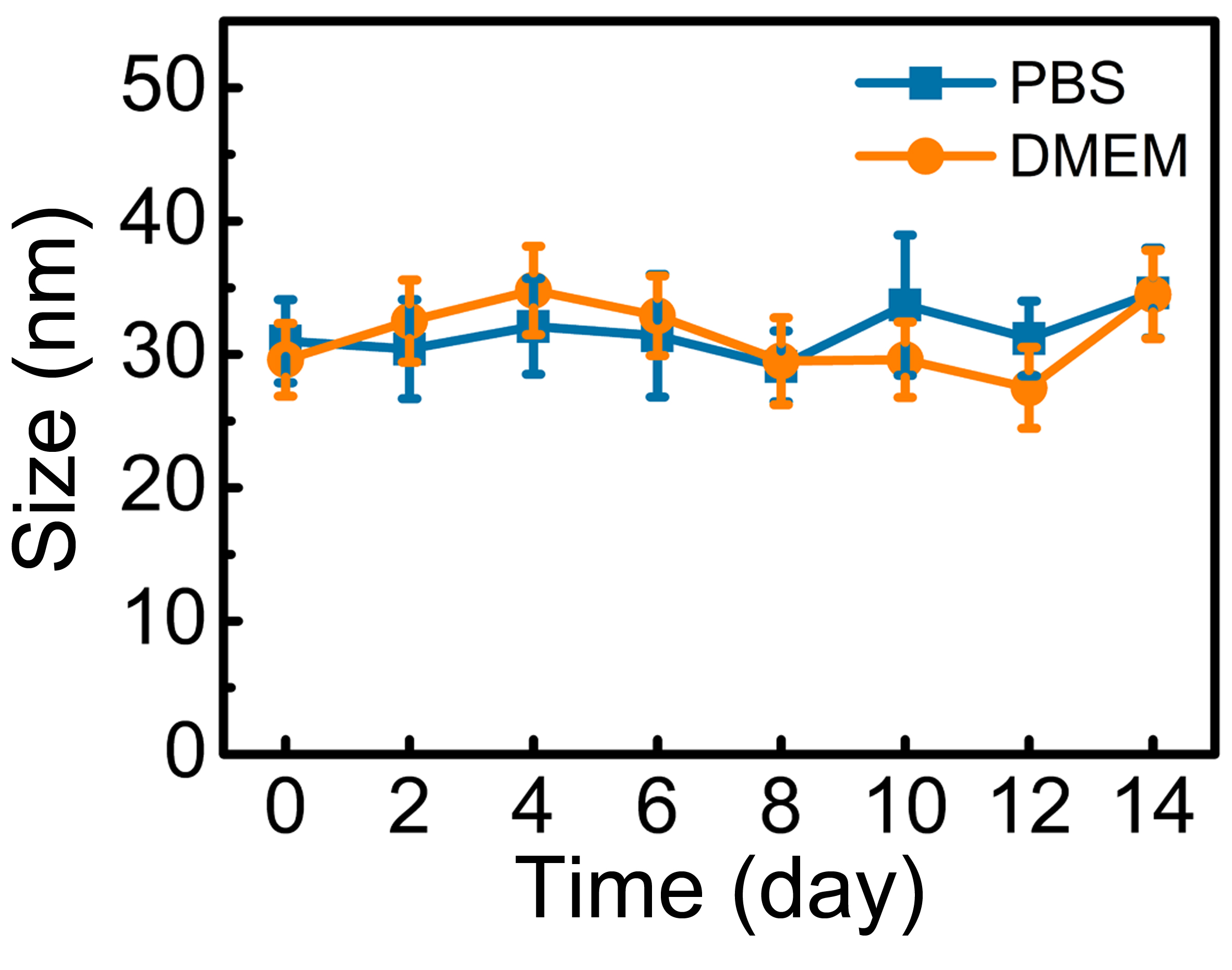


**Figure S6.** Hydrodynamic size changes of EM NSs dispersed in PBS or DMEM for a duration of two weeks as measured by DLS.


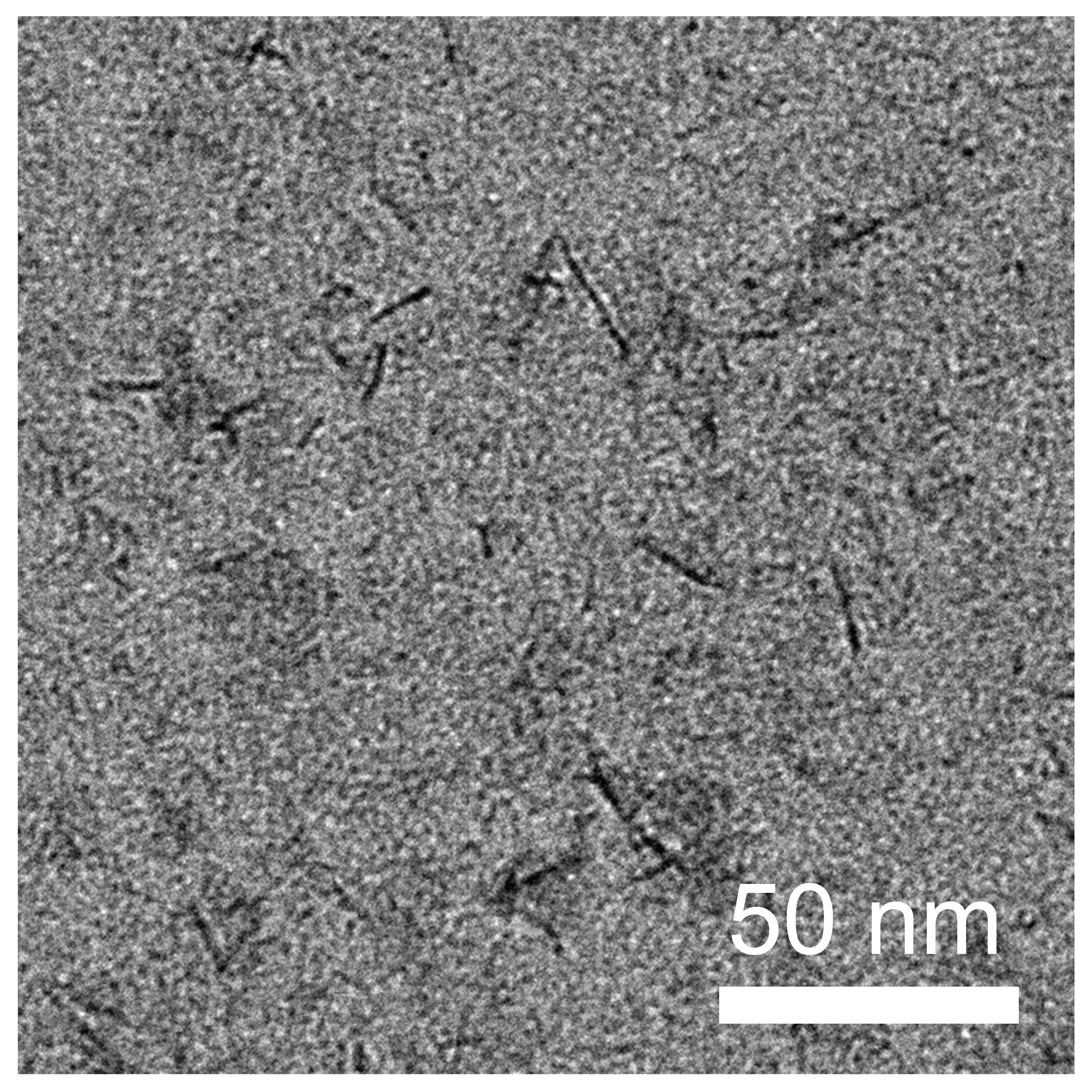


**Figure S7.** Enlarged version of Figure 3(a) for clearer observation.


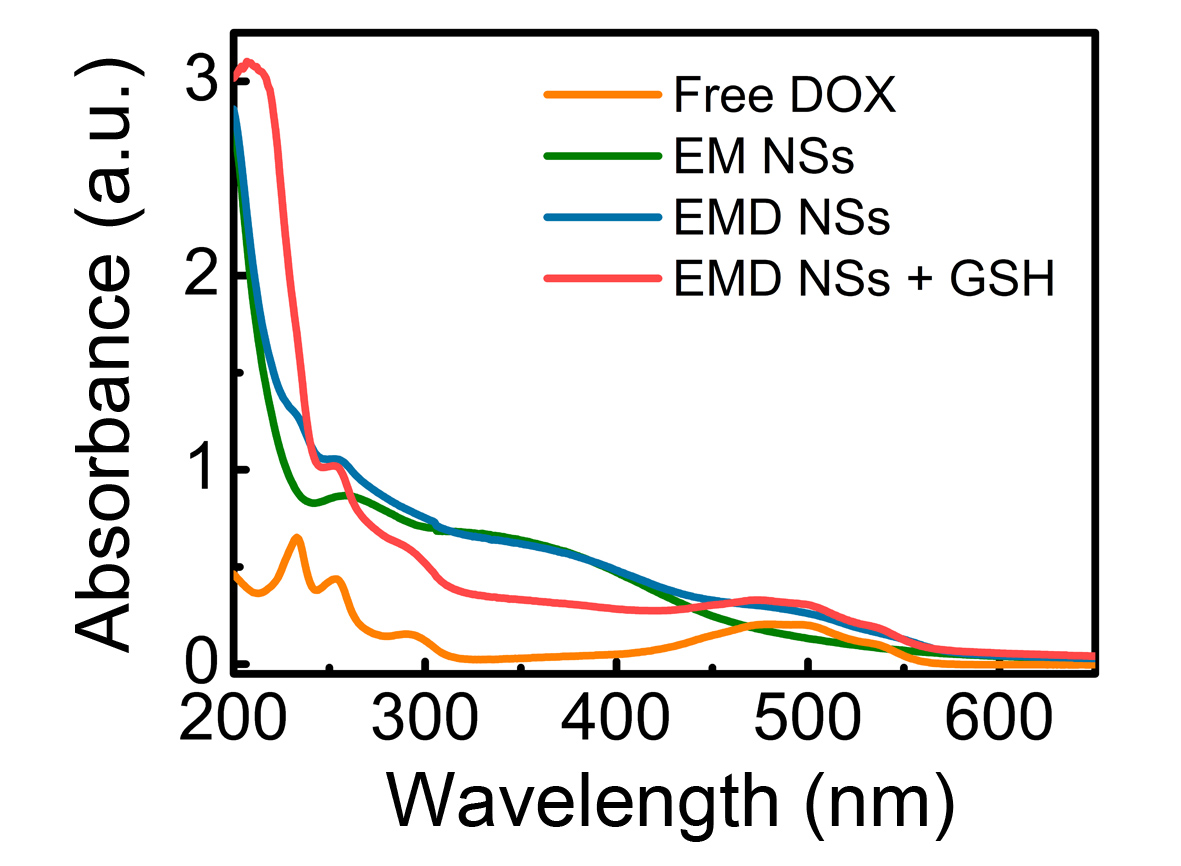


**Figure S8.** UV–vis absorption spectra of aqueous solutions containing free DOX, EM NSs, EMD NSs, or EMD NSs + GSH.


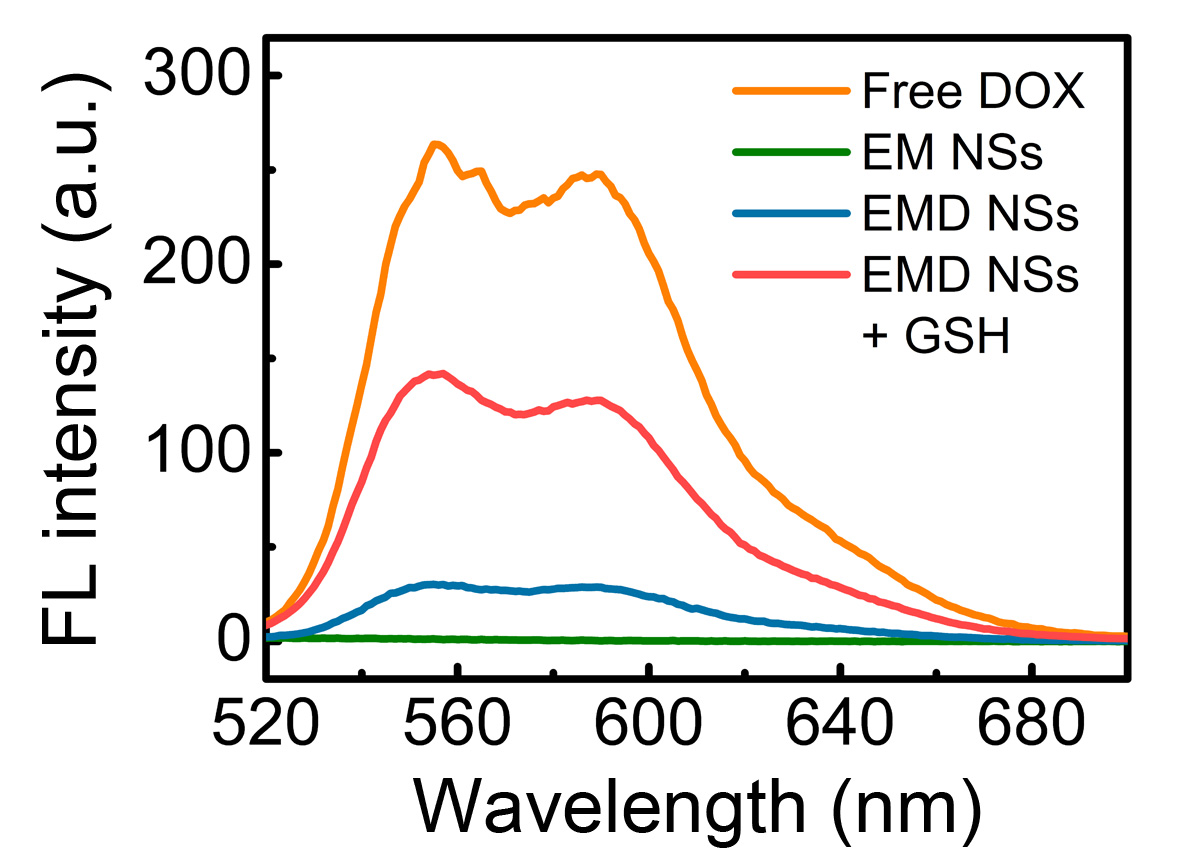


**Figure S9.** FL emission spectra of aqueous solutions containing free DOX, EM NSs, EMD NSs, or EMD NSs + GSH. *Ex* = 500 nm.


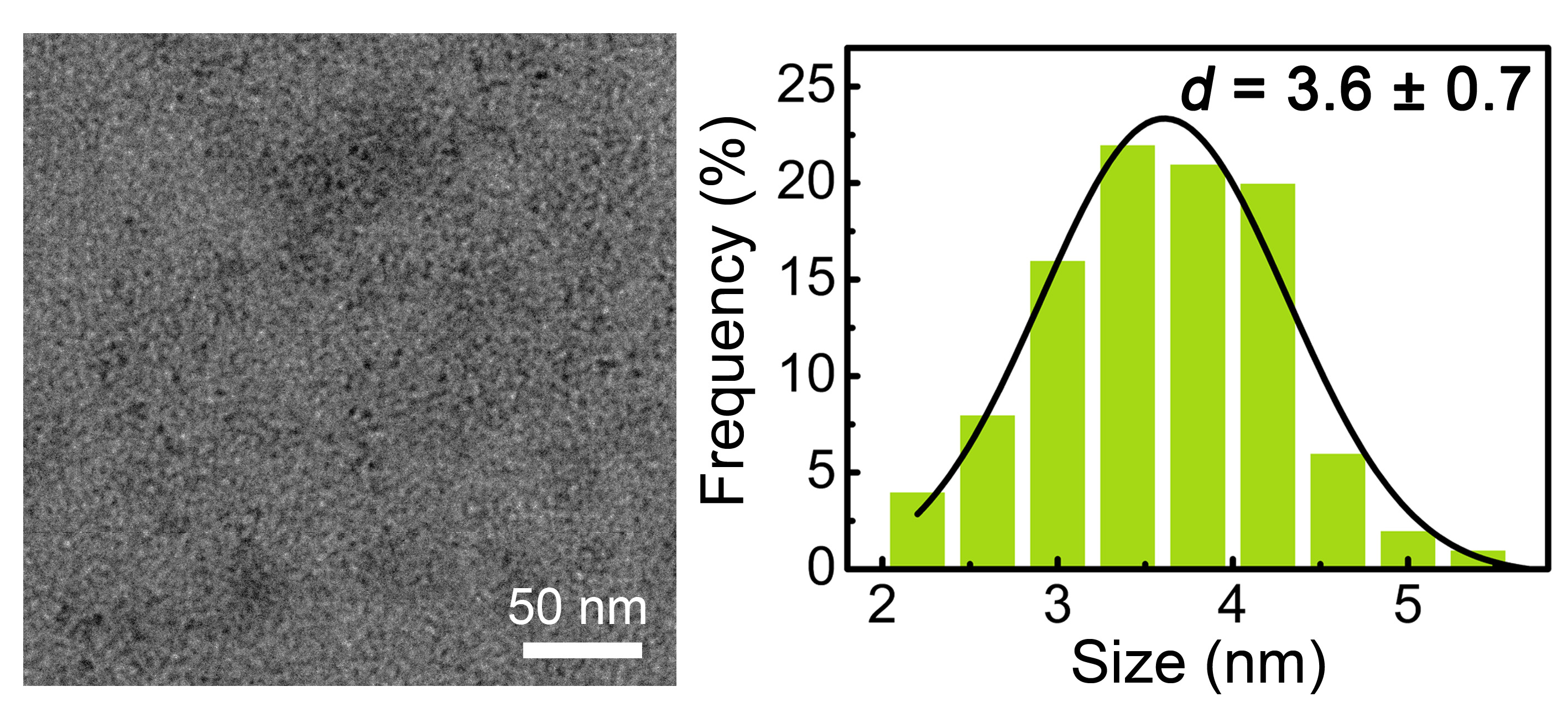


**Figure S10.** TEM image (left) and corresponding size histogram (right) of EM NSs after treatment with GSH (2.0 mM).


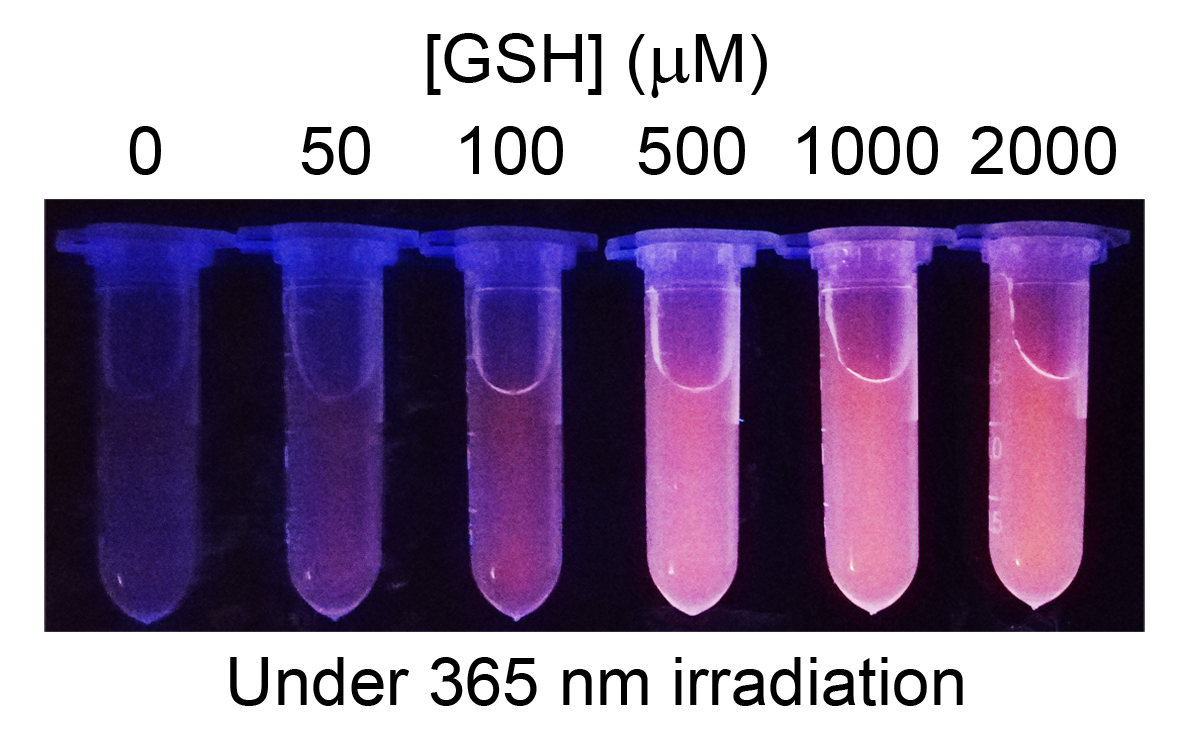


**Figure S11.** Photographs of EMD NS suspensions after mixing with various concentrations of GSH under a UV lamp (365 nm excitation). The final GSH concentrations are indicated in the figure.


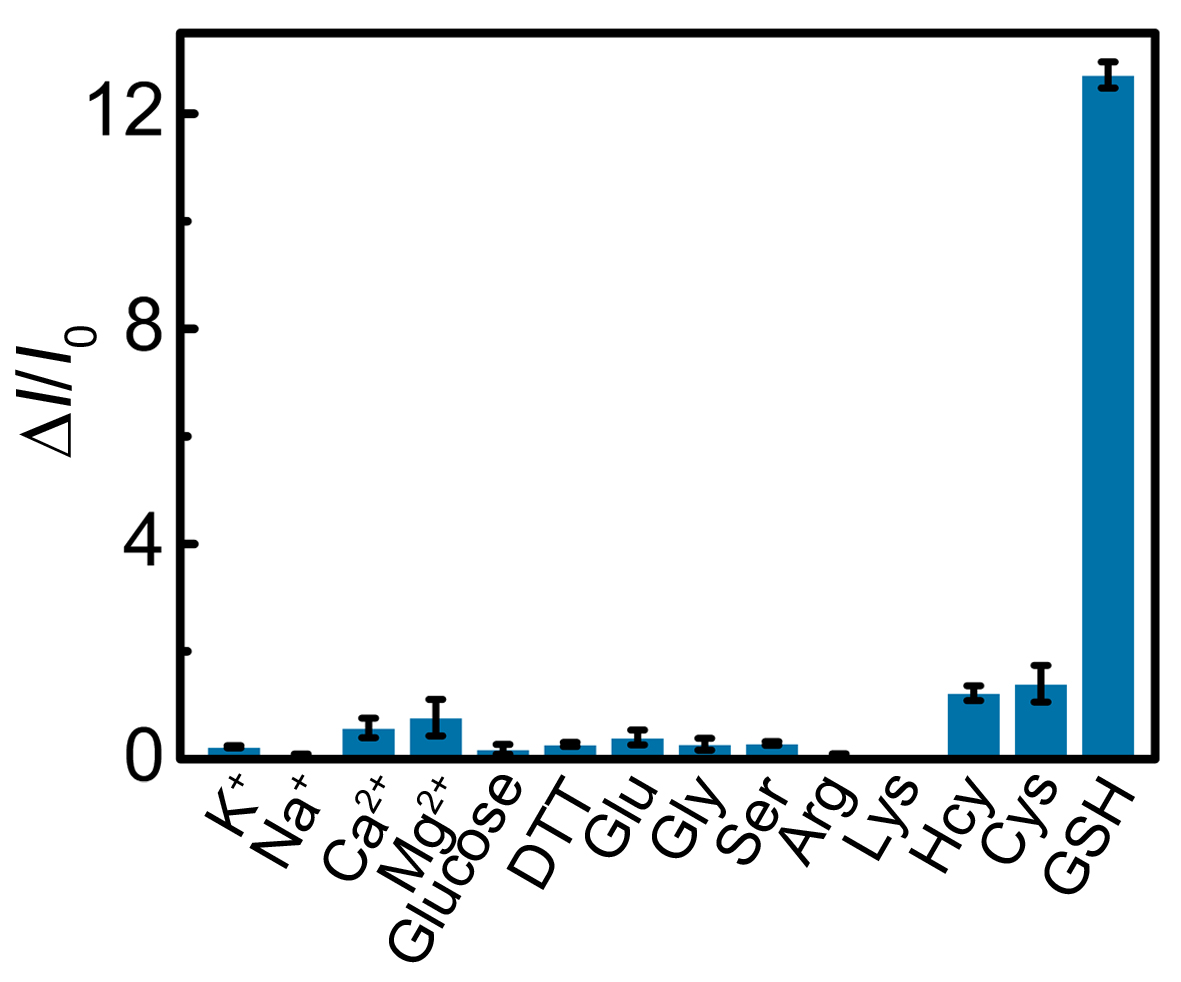


**Figure S12.** FL responses of EMD NSs to different guest molecules. The concentrations of all guests were 1.0 mM.


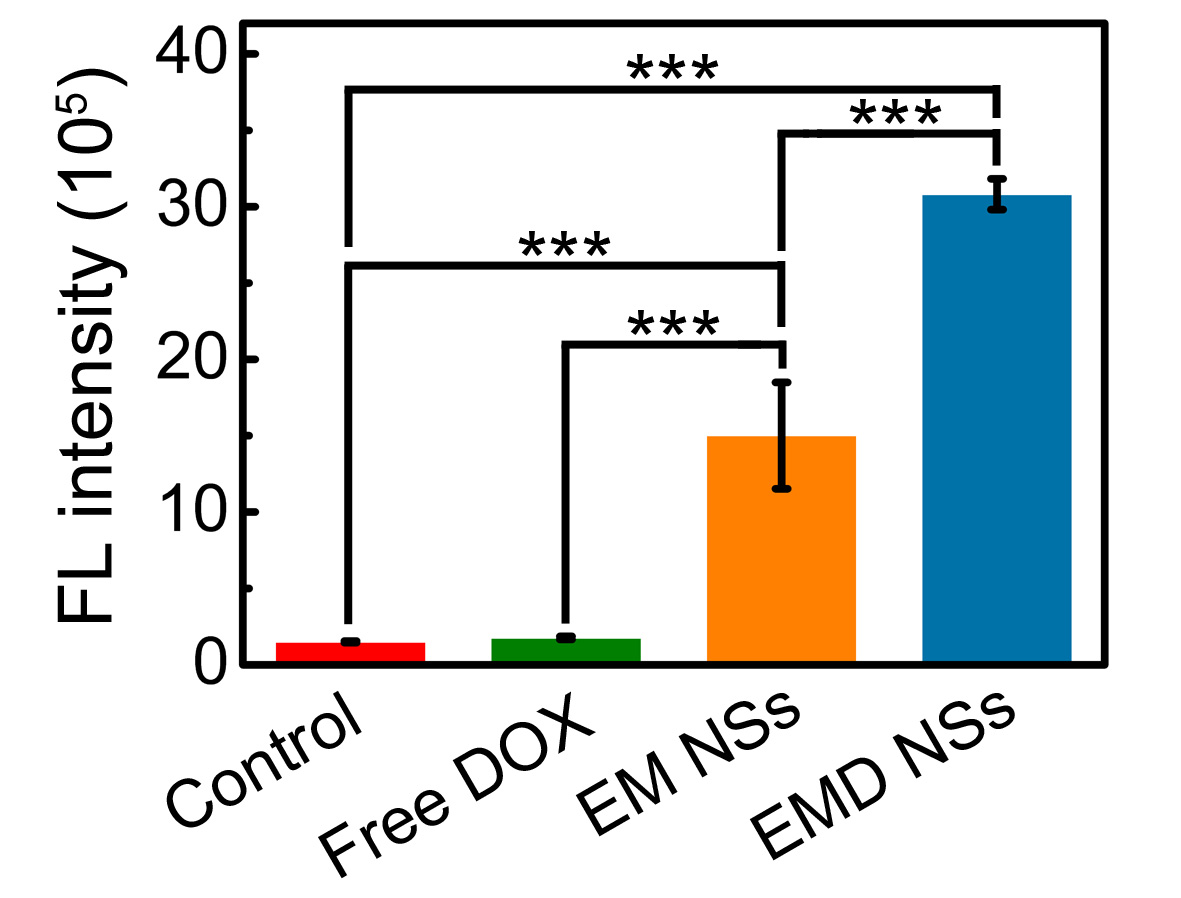


**Figure S13.** Flow cytometry-based FL analysis of cellular ROS levels after various treatments as indicated. ****P* < 0.001.


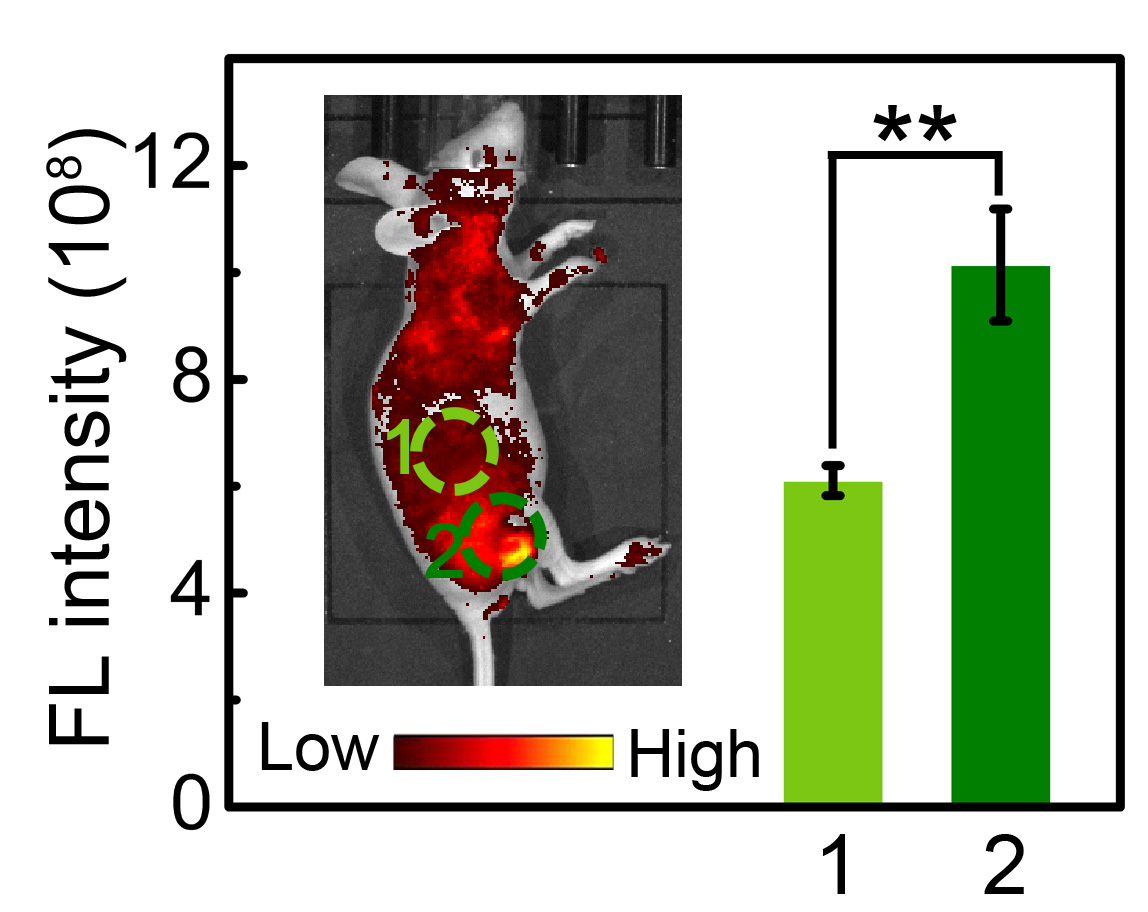


**Figure S14.** In vivo FL image (inset) and corresponding FL analysis result of a mouse injected with EMD NSs. “1” and “2” indicate the normal and tumor areas injected with EMD NSs, respectively. ***P* < 0.01.


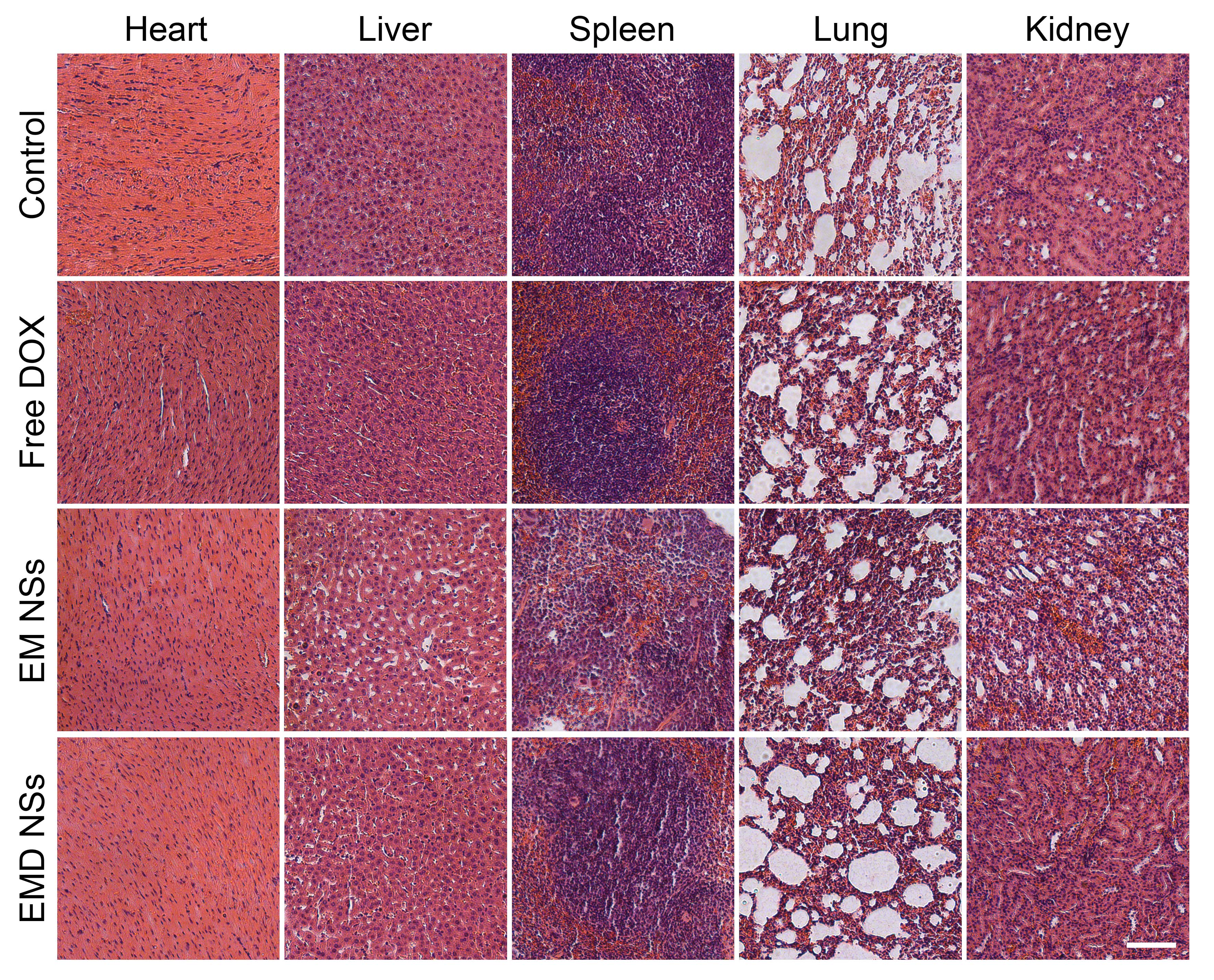


**Figure S15.** Histological analysis of major organs collected from mice sacrificed on the 14th day post i.v. injection with PBS (control), free DOX, EM NSs, or EMD NSs. Scale bar = 100 µm.


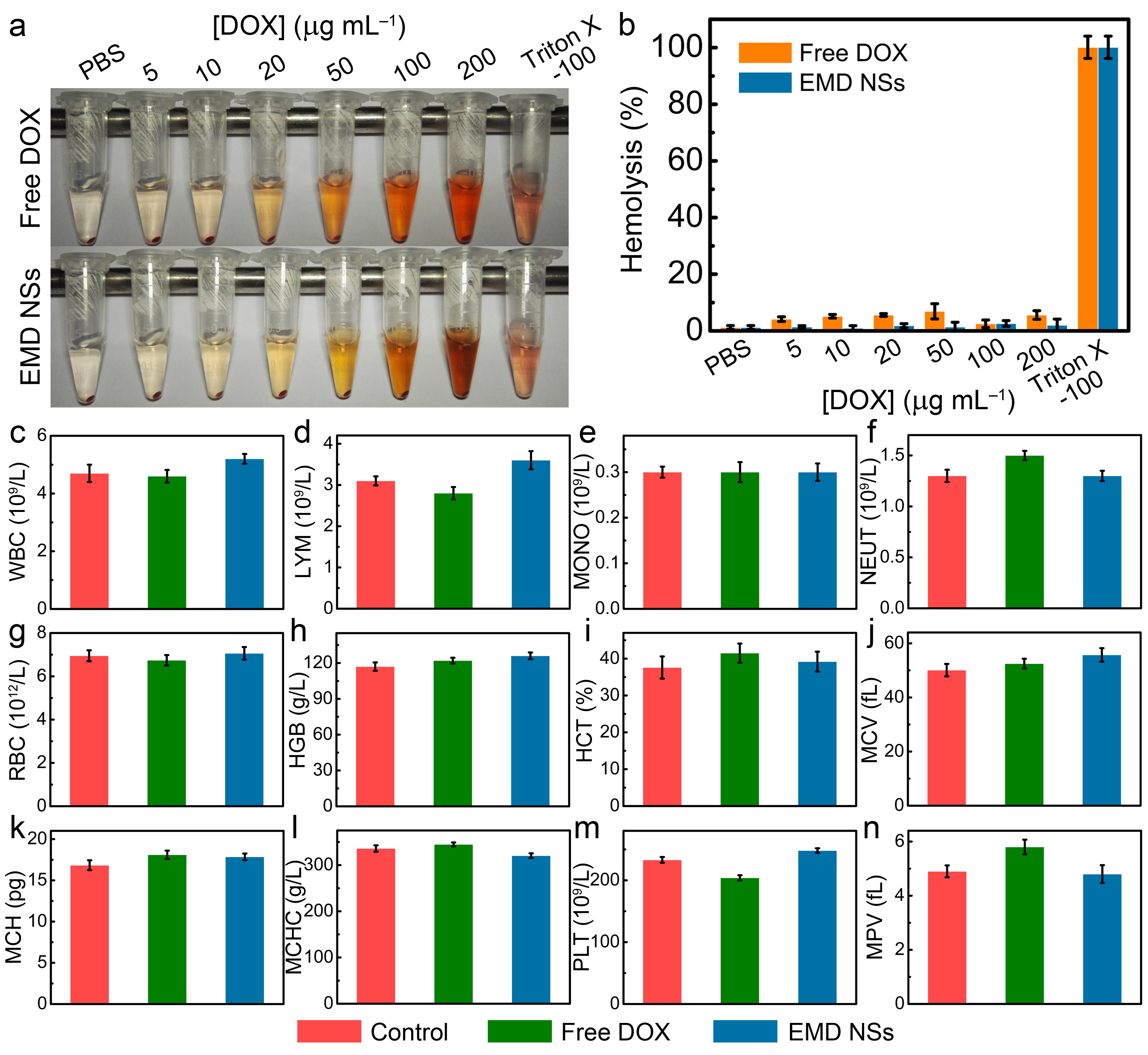


**Figure S16.** (a) Photographs of RBC suspensions after hemolysis assay. (b) Hemolysis percentages of RBCs treated with PBS (negative control), and Triton X-100 (positive control), or varied concentrations of free DOX or EMD NSs. DOX concentration was abbreviated as [DOX] in (a and b). (c–n) Blood routine analysis results of mice after treatment with PBS (control), free DOX, or EMD NSs. The blood indexes including WBC, LYM, MONO, NEUT, RBC, HGB, HCT, MCV, MCH, MCHC, PLT, and MPV indicate numbers of white blood cells, lymphocytes, monocytes, neutrophilic granulocytes, and red blood cells, concentration of hemoglobin, hematocrit, mean corpuscular volume, mean corpuscular hemoglobin, mean corpuscular hemoglobin concentration, platelet count, and mean platelet volume, respectively.
